# Targeted NMDA Receptor Interventions for Autism: Developmentally Determined Expression of GluN2B and GluN2A-Containing Receptors and Balanced Allosteric Modulatory Approaches

**DOI:** 10.3390/biom12020181

**Published:** 2022-01-22

**Authors:** Stephen I. Deutsch, Zachary N. M. Luyo, Jessica A. Burket

**Affiliations:** 1 Department of Psychiatry and Behavioral Sciences, Eastern Virginia Medical School, 825 Fairfax Avenue, Suite 710, Norfolk, VA 23507, USA; deutscsi@evms.edu; 2 Program in Neuroscience, Christopher Newport University, Newport News, VA 23606, USA; zachary.luyo.18@cnu.edu; 3 Department of Molecular Biology & Chemistry, Christopher Newport University, Newport News, VA 23606, USA

**Keywords:** NMDA receptor, autism spectrum disorder, positive allosteric modulators, negative allosteric modulators

## Abstract

Various ASD risk alleles have been associated with impairment of NMDA receptor activation (i.e., NMDA Receptor Hypofunction) and/or disturbance of the careful balance between activation mediated by GluN2B-subtype and GluN2A-subtype-containing NMDA receptors. Importantly, although these various risk alleles affect NMDA receptor activation through different mechanisms, they share the pathogenic consequences of causing disturbance of highly regulated NMDA receptor activation. Disturbances of NMDA receptor activation due to sequence variants, protein termination variants and copy number variants are often cell-specific and regionally selective. Thus, translational therapeutic NMDA receptor agonist interventions, which may require chronic administration, must have specificity, selectivity and facilitate NMDA receptor activation in a manner that is physiologic (i.e., mimicking that of endogenously released glutamate and glycine/D-serine released in response to salient and relevant socio-cognitive provocations within discrete neural circuits). Importantly, knockout mice with absent expression and mice with haploinsufficient expression of the deleterious genes often serve as good models to test the potential efficacy of promising pharmacotherapeutic strategies. The Review considers diverse examples of “illness” genes, their pathogenic effects on NMDA receptor activation and, when available, results of studies of impaired sociability in mouse models, including “proof of principle/proof of concept” experiments exploring NMDA receptor agonist interventions and the development of promising positive allosteric modulators (PAMs), which serve as support and models for developing an inventory of PAMs and negative allosteric modulators (NAMs) for translational therapeutic intervention. Conceivably, selective PAMs and NAMs either alone or in combination will be administered to patients guided by their genotype in order to potentiate and/or restore disrupted balance between activation mediated by GluN2B-subtype and GluN2A-subtype containing NMDA receptors.

## 1. Introduction

Currently, approved medication strategies for autism spectrum disorder (ASD) target secondary symptoms of agitation and irritability. Clinicians will often employ a variety of medications from different pharmacological classes, whose effectiveness is variable and indications are uncertain, to address comorbid anxiety, disturbances of mood, psychotic symptoms, repetitive motor behaviors, obsessive thinking, and cognitive inflexibility. However, as risk alleles are identified and their potential pathogenic consequences elucidated, it is hoped that medication strategies will be developed to target the etiopathogenic basis of core symptom domains in genotypically distinct types of ASD, such as deficits in social communication and social interaction (i.e., social communication) and “restricted, repetitive patterns of behavior, interests, or activities”.

Abnormalities of NMDA receptor-mediated neurotransmission have been implicated in presentations of autism spectrum disorder (ASD) associated with a variety of risk alleles. Perhaps, not surprisingly, sequence variants of the heterotetrameric NMDA receptor have been identified with “next-generation sequence technologies” that are associated with neurodevelopmental phenotypes that include combinations of variably expressed ASD, developmental delay and intellectual disability, epilepsy, alterations of muscle tone (i.e., both hypo- and hypertonia), language problems, and dysmorphic features, among other phenotypic manifestations [1]. Increasingly, rare and novel sequence variants are being identified in individual patients with ASD and other neuropsychiatric disorders, including missense mutations that result in single amino acid substitutions in critical regions of the C-terminus, with high-throughput third-generation deep sequencing. These human missense mutations lead to studies of the homologous mouse and rat missense mutations expressed in cell cultures and genetically engineered animal models in an effort to clarify their effects on synaptic targeting and surface expression, intracellular binding with interacting binding partners, single channel function, neural circuits, and behavior. The proposed pathogenic risk alleles are often “loss or gain of function” sequence variants, protein termination variants, or identified as candidate loci within spans of chromosomal microdeletions (i.e., copy number variants (CNVs)), among other possible mutations. In addition to their etiological involvement in causing NMDA receptor hypofunction (NRH), pathological consequences of some of these risk alleles include disruption of a finely tuned balance between activation of glutamate-gated ion conductance mediated by GluN2B-subtype and GluN2A-subtype-containing NMDA receptors. In some instances, mouse models with absent (i.e., knockout (KO) mice) or haploinsufficient expression of the implicated genes have been created and show impaired social behaviors that improve with administration of targeted NMDA receptor agonist interventions. Ultimately, an appreciation of the phenotypic consequences of specific sequence variants will depend on a synthesis of data regarding their developmental expression, anatomic distribution, complex effects on single-channel electrophysiology, and impact of expression in single neurons on the neural circuitry in which these single neurons expressing mutated receptors are embedded [1]. As stated, these sequence variants can be associated with gain or loss of function, and their phenotypic effects may reflect dominant negative mechanisms of genetic expression. 

Targeting orthosteric sites that bind glutamate lack spatial and cell type selectivity because salient features of the electronic configuration of agonist recognition sites for glutamate are shared across a variety of ionotropic and metabotropic glutamate receptors, and NMDA receptors themselves are diffusely distributed throughout the brain. Thus, the obligatory co-agonist binding site for glycine and D-serine emerged as a preferred target in several “proof of principle/proof of concept” exploratory preclinical studies and early phase clinical trials, whose aim was to facilitate NMDA receptor activation [2,3,4,5]. Targeting the obligatory co-agonist binding site improves selectivity for NMDA receptors and avoids off-target stimulation of other classes of glutamate ionotropic and metabotropic receptors. Tools and potential strategies for targeting the obligatory co-agonist binding site have included and include administration of the following: an acylated glycine prodrug; a partial glycine agonist; sarcosine-derived and non-sarcosine-derived inhibitors of the glycine transporter type 1 (GlyT1); and inhibitors of D-amino acid oxidase (DAAO), the latter enzyme catabolizes D-serine [5,6,7,8]. However, as pharmacotherapeutic interventions for ASD and other developmental disorders (DD) are likely to be long-term, therapeutic strategies that mimic spatial (i.e., targeting NMDA receptors expressed on the surface of specific neurons found within specific neural circuits that serve higher socio-cognitive functions) and temporal (i.e., mimic physiological patterns of irregular firing and release) selectivity of endogenous ligands are sought. Thus, interest in the development of positive and negative allosteric modulators (i.e., PAMs and NAMs, respectively) is increasing. 

Conceivably, selective PAMs and NAMs either alone or in combination will be administered to patients guided by their genotype in order to potentiate and/or restore disrupted balance between activation mediated by GluN2B-subtype and GluN2A-subtype containing NMDA receptors [9,10,11,12]. The challenges are to understand the implications and impact of these expressed individual sequence variants on alterations of: conductance and gating properties of the ion channel and binding properties of the glutamate and glycine/D-serine orthosteric binding sites; pharmacological properties of allosteric modulatory sites [13]. Metabotropic functions of the resulting assembled heterotetrameric channels may also relate to both efficiency of Ca^2+^ conductance and the binding of interacting partners to the C-terminal cytoplasmic tails of receptor subunits [13]. These challenges are confounded by several factors including the developmental regulation of expression of NMDA receptor subunits and non-uniform distribution and anatomically selective enrichment of expression of assembled NMDA receptors, whose subunit compositions can reflect different combinations (e.g., two obligatory GluN1 subunits with either two identical or dissimilar GluN2 subunits) [1,13,14,15,16,17,18]. Differences with respect to synaptic and extrasynaptic expression and “preference” for binding either the glycine or D-serine co-agonist to these synaptic and extrasynaptic receptors also contribute to this complexity [1,13,14,15,16,17,18]. Although encoded by a single gene, the obligatory GluN1 subunits have eight RNA splice variants, and there are four distinct genes coding different GluN2 subunits (i.e., *GRIN2A*, *GRIN2B*, *GRIN2C* and *GRIN2D*); again, the expression of these distinctive subunits in assembled channels shows anatomic selectivity, developmental regulation, and combinatorial diversity [1,13].

Unfortunately, translational development of clinically useful medications must await continued development of a large inventory of selective PAMs and NAMs that can be administered safely and chronically to patients. In order to increase the probability of “disease-modifying” effects on the trajectories of cognitive and social development of affected patients, these medications may need to be started early and continued at least through early adulthood. Development of pharmacotherapeutic strategies will be facilitated by expression of specific sequence variants in transgenic mice, and characterization of their effects on relevant behavioral readouts, such as social preference and memory, measures of cognition, changes in threshold for chemical and electrically evoked seizures, among other relevant behavioral paradigms that measure locomotor activity, anxiety, and mood [7,19,20,21,22,23]. The “hope” is that going from the “bedside to the bench” will not only clarify the mechanisms of pathogenesis, but also inform novel and more effective pharmacotherapeutic strategies [24].

This Review considers diverse examples of “illness” genes, their pathogenic effects on NMDA receptor activation (Table 1) and the results of studies of impaired sociability in mouse models, including “proof of principle/proof of concept” experiments exploring NMDA receptor agonist interventions. Moreover, we will provide discussion on the development of promising representative PAMs, which serve as support and models for developing an inventory of PAMs and NAMs for translational therapeutic intervention and provide an update of other developmental efforts in this area [9]. Importantly, there is a moral imperative to develop newer medication strategies that will complement other essential components of individualized, multimodal treatment plans because current clinical outcomes of patients with ASD are less than optimal and both patients and “unaffected” neurotypical family members experience much morbidity.

## 2. Convergence of Various Diverse Genetic Lesions to Disrupt NMDAR-Mediated Neurotransmission: Influences on NMDAR Hypofunction and Imbalance between GluN2B and GluN2A-NMDAR-Mediated Activation

### 2.1. 16p11.2, a Genetic Lesion Resulting in NMDA Receptor Hypofunction

The 16p11.2 locus on the proximal short arm of human chromosome 16 spanning 500 to 600 kb and containing 27 to 29 genes is a “hotspot” for copy number variants (CNVs) [25,26]. Microdeletions of 16p11.2 are commonly associated with ASD: about 0.5% of ASD cases are found to have this deletion and about 16% to 26% of carriers of the deletion are diagnosed with ASD. CNVs, including both heterozygous deletions and duplications, are associated with variable phenotypic expression, including ASD, intellectual disability, and schizophrenia [26]. Interestingly, the 11.2 position on the short arm of chromosome 16 contains genes implicated in abnormalities of cortical development (e.g., *MAPK3*, *KCTD13*, and *TAOK2*). 

Of interest to the current Review, mice heterozygous for the deletion (*16p11*^+/−^) showing behavioral deficits of impaired spatial memory and impaired social motivation have diminished electrically evoked NMDA receptor-activation of excitatory postsynaptic currents (NMDAR-EPSCs) in layer 5 pyramidal neurons of medial prefrontal cortex (mPFC) [25]. This NMDA receptor hypofunction in *16p11*^+/−^ mice was not due to the diminished protein expression of GluN2B or GluN2A NMDA receptor subunits in synaptic membranes prepared from frontal cortex tissue, but appeared to be due to the diminished phosphorylation of the S1303 residue on the GluN2B receptor subunit. In support of the diminished phosphorylation of S1303 contributing to the pathogenic effect, a “chemogenetic” strategy was adopted to phosphorylate the GluN2B subunit. This was achieved via viral delivery of a “designer receptor exclusively activated by designer drug (DREADD)” to the prefrontal cortex of *16p11*^+/−^ mice, which was selectively activated by clozapine-*N*-oxide (CNO), that drove CaMKII-mediated phosphorylation of the S1303 subunit. Data show that behavioral deficits were rescued and the amplitude of electrically evoked NMDA receptor-activated EPSCs in mPFC were normalized [25]. 

Specifically, CaMKII-mediated phosphorylation of the S1303 site on the GluN2B subunit as a downstream consequence of G_q_ signaling seemed to normalize NMDA receptor hypofunction. Intraperitoneal injection of CNO increased the frequency of recorded spontaneous action potentials in slices obtained from the DREADD-injected *16p11*^+/−^ mice, restoring this measure of neuronal activity to levels observed in slices from wild type mice. Similarly, chemogenetic activation increased NMDAR-EPSCs in PFC pyramidal neurons from DREADD-injected *16p11*^+/−^ mice; chemogenetic activation also raised the amplitudes of NMDAR-EPSCs in the DREADD-injected *16p11*^+/−^ mice to levels observed in the wild type slices [25]. Further, CNO treatment significantly increased phosphorylation of the GluN2B subunit at its S1303 site in lysates of PFC slices obtained from the DREADD-injected *16p11*^+/−^ mice. The chemogenetic activation rescuing NMDA receptor hypofunction associated with increased GluN2B phosphorylation at the S1303 site also had positive behavioral consequences in the *16p11*^+/−^ mice [25]. 

Both spatial working memory measured in the Barnes maze and social approach/interaction with an enclosed stimulus mouse (both the time spent interacting and the number of interactions) improved after chemogenetic activation of the DREADD-injected *16p11*^+/−^ mice. Overall, these data suggest that NMDA receptor hypofunction can be a downstream consequence of subtle disturbances in the “architecture” of the developing brain and support translational therapeutic strategies to promote NMDA receptor-mediated neurotransmission for at least some presentations of ASD.

### 2.2. Cullin 3 Deficiency, Association with NMDA Receptor Hypofunction

Increasingly, because of extensive cross-talk between signaling and metabolic pathways, there is recognition that NMDA receptor hypofunction and ASD psychopathology can result from defects unrelated to sequence variants of the NMDA receptor subunits themselves. A case in point is the gene encoding Cullin 3 (Cul3), a component of the E3 ubiquitin ligase complex that is a high-risk allele for autism and schizophrenia [27]. Large scale unbiased genetic screening identified recurrent de novo heterozygous loss of function mutations of *Cullin 3* (*Cul3*) in cohorts of patients with autism. A recent investigation explored molecular, electrophysiological and behavioral consequences of Cul3 deficiency in forebrain-specific *Cul3* knockout mice [27]; a major finding was the presence of NMDA receptor hypofunction in prefrontal cortex with obvious translational implications for treatment. In addition to studying Cul3 deficiency in the forebrain, *Cul3* deficiency was also studied when it was knocked out with anatomic selectivity in prefrontal cortex and striatum in order to exam multilevel impacts of its deficient expression on ASD psychopathology because cortico-striatal alterations are involved in the pathophysiology of autism and schizophrenia. In addition to Cul3′s role in processing substrates for ubiquitination and proteosomal degradation, it is also involved in cytoskeletal organization and cell differentiation. Additionally, one of Cul3′s “adapters” is coded by chromosome 16p11.2, a locus that is subject to microdeletions and microduplications (i.e., copy number variants), and associated with autism and schizophrenia.

Mice with heterozygous forebrain deletion of *Cul3* spent less time in the chamber housing a social stimulus mouse and showed no significant preference for an enclosed mouse over an empty inverted enclosure in the three-chamber sociability apparatus [27]. Importantly, when virally mediated selective knockout of *Cul3* was achieved bilaterally in prefrontal cortical pyramidal neurons, the mice with these selective regional and cell-specific genetic lesions also spent significantly less time interacting with social stimulus mice and less time in the chamber containing enclosed stimulus mice than control virally injected mice [27]. The impaired sociability was related to the specific region and cell-type undergoing virally mediated knockout of *Cul3* because bilateral virally mediated knockout of *Cul3* in dorsal striatum that significantly reduced expression of *Cul3* in striatal medium spiny neurons had no effect on social preference in the three-chamber sociability apparatus, but did increase repetitive grooming, compared to virally injected control mice [27]. (Repetitive stereotypic behavior is a hallmark of ASD psychopathology.) Whole-cell patch-clamp recordings showed that amplitudes of electrically evoked NMDA receptor-mediated excitatory postsynaptic currents (EPSCs) in frontal cortical pyramidal neurons of mice with forebrain-specific *Cul3* deletions were significantly reduced compared to controls [27]. The NMDA receptor hypofunction in frontal cortex appeared to be due to reduced expression of the obligatory GluN1 subunit in mice with forebrain-deficient expression of *Cul3*.

Gene ontology analyses and proteomic studies suggested that at least some of the consequences of forebrain-deficient *Cul3* expression relate to upregulated expression of Smyd3, a protein regulating the methylation status of specific lysine residues in the histone H3 protein, in forebrain of Cul3-deficient mice; methylation status of histones influences transcriptional efficiency [27]. Thus, it is of translational interest that BCI-121, a small molecule Smyd3 inhibitor that reduces H3K4 di- and tri-methylation and downregulates Smyd3-targeted effects on gene transcription, increased NMDA receptor-mediated synaptic currents in prefrontal cortex and improved social preference and time spent interacting with social stimulus mice in mice with forebrain-deficient expression of *Cul3* [27]. The data clearly implicate NMDA receptor hypofunction in prefrontal cortex as a “downstream” consequence of Cul3-deficiency and potential driver of impaired sociability, a major domain of ASD psychopathology [4,21,28].

From a translational therapeutic perspective, targeting NMDA receptor hypofunction may improve information transfer within anatomically discrete circuits with derivative therapeutic benefit on cognitive and complex socio-cognitive functions, and that dampening NMDA receptor-mediated neurotransmission may be a shared mechanism of ASD pathogenesis associated with a variety of genetic sequence variants and copy number variants [4,5,7,13,29,30,31,32].

### 2.3. M2 Pore Loop Variants

Complex electrophysiological consequences of 12 discrete de novo missense mutations in the M2-reentrant loop of *GRIN1*, *GRIN2A*, and *GRIN2B* loci, described in 18 patients with variable phenotypes, were characterized [1]. The phenotype of all 18 patients included developmental delay and intellectual disability; half of the patients had early onset of a variety of seizure types (including infantile spasms, focal seizures, myoclonus, generalized tonic clonic seizures, and status epilepticus); and a variable number (from 2 to 11 patients) had “autistic features”, altered muscle tone, language problems and dysmorphic features [1]. These missense mutations in the second half of the M2 reentrant poor loop were subject to “strong purifying selection”, which is consistent with the greater likelihood that their occurrence is “harmful”; and more likely to originate de novo. The fact missense mutations in the second half of the M2 pore loop of *GRIN1*, *GRIN2A*, and *GRIN2B* genes were rare in a reference genomic database interrogated in mid-January 2019 is consistent with their harmful effects and greater likelihood they derive de novo [1]. The authors noted that the 12 patient-ascertained de novo M2 pore loop variants considered by them reside on the pore-forming side of the reentrant loop, which limit generalizations of their findings to variant missense mutations that face the pore, but mutagenesis studies suggesting that mutations that do not face the pore also affect ion permeation and Mg^2+^ blockade were cited [1].

An “overall” effect of heterozygous expression of single copies of representative missense mutations coding for M2 pore-facing loop variants was a decreased sensitivity of assembled receptors containing even single copies of mutated subunits to Mg^2+^ blockade [1]. This diminished Mg^2+^ blockade suggests that the majority of missense *GRIN* M2 pore loop variants will be “gain-of-function” mutations. However, although the overall effect will be enhanced NMDA receptor function, individual dimensions of the electrophysiological response to specific missense mutations vary with some having directional effects opposed to enhanced NMDA receptor activation. Receptors assembled from mutated transcripts were associated with specific effects on channel function that differed from expression of the wild type transcript. For example, responses of “two-electrode voltage-clamp (TEVC) recordings from *Xenopus laevis* oocytes” to agonist activation by glutamate and glycine and sensitivity to proton concentrations, as determined by recordings of current response amplitudes obtained at pH 6.8 and pH 7.6, differed as a function of representative GluN1, GluN2A and GluN2B variants versus receptors assembled from expressed wild type transcripts. Moreover, M2 pore loop variants affected deactivation response time in response to rapid removal of agonist, which controls the time course of the slow component of the excitatory postsynaptic current (EPSC); single channel open probability; Ca^2+^ permeability; and surface expression of assembled receptors. Nonetheless, “functional enhancements” resulting from the decreased sensitivity to Mg^2+^ blockade compensate and favor receptor activation by agonists, overriding effects of the missense mutations that favor decreased current responses. However, the authors caution against an over-simplified “binary classification” that characterizes mutations as possessing “overall gain- or loss of function” [1]. Importantly, specific effects of M2 pore loop variants in the local microenvironments of specific neurons integrated into specific circuits, such as altered deactivation kinetics or altered proton sensitivity of NMDA receptors, especially in the context of pathological changes that affect local acid–base balance or ion buffering, may influence phenotypic manifestations and severity of signs/symptoms and the efficacy of proposed pharmacotherapeutic interventions [1,33].

### 2.4. Intracellular Proteins and Signaling Mechanisms Contributing to NMDA Receptor Regulation

More than 2000 proteins have been identified within the postsynaptic density (PSD) located at the postsynaptic membrane of excitatory synapses [34]. Scaffolding proteins are core components of the PSD, whose individual multi-domain architectures serve as platforms for binding with multiple interacting partners and are responsible for their specialized protein–protein interactions (PPIs). The PSD, a complex morphological structure, is an end-result of aligning these interacting binding partners (i.e., scaffolding proteins) into three layers of protein–protein interacting networks (PINs). The top layer forms along the cytoplasmic side of the cell surface consisting of DLG proteins with PDZ binding domains that bind to cytoplasmic tails of glutamate and other neurotransmitter receptors, while the family of Shank proteins with their ‘SH3 and multiple Ankyrin repeat domains’ are at the bottom layer; the top and bottom layers are connected by a middle layer of ‘DLG-associated scaffolding proteins (DLGAP1-4)’ [34]. Multiple domains of individual proteins within each of the three layers permit their binding to a variety of interacting partners creating bidirectional macromolecular signaling complexes, wherein communication flows back and forth between receptors at the cell surface, a variety of enzymes (e.g., calcium-calmodulin-dependent protein kinases) and cytoskeletal structural proteins. Importantly, these large “three-layered” macromolecular signaling complexes are not static, but dynamically regulated in response to such things as developmental cues and signaling events at the cell surface, among other regulatory influences [34]. The numerous “combinations” of potential interacting protein partners with each other and the multiple PPIs with which any individual protein can engage support roles for these bidirectional macromolecular signaling complexes in “simultaneous regulation of multiple signaling pathways and cellular processes” [34]. Again, studies reveal differences in composition of core-scaffolding proteins as a function of species, stage of development, anatomic brain region, tissue fractionation techniques, cell types (including differences between dendrites and soma), and environmental influences (e.g., synaptic differences related to learning, memory and synaptic plasticity). Increasingly, there is interest in genetically determined pathogenic changes of the “postsynaptic interactome” in neuropsychiatric disorders, including ASD, which may occur as a result of single nucleotide polymorphisms (SNPs) identified in Genome Wide Association Studies (GWAS), de novo single nucleotide variants (SNVs), and copy number variants (CNVs) [34].

#### PSD95/SAP90/DLG4

The intracellular C-terminal region of the GluN2B receptor subunit is critical for the intracellular trafficking of GluN2B-containing NMDA receptors and their targeted insertion at synaptic sites on the cell surface, as well as their alignment with and binding to critical interacting partners, such as the multidomain scaffolding protein, PSD-95, and other members of the “membrane-associated guanylate kinase (MAGUKs)” family of proteins [24]. In particular, the last four amino acids of the GluN2B C-terminus (a.a. 1479-1482) act as a PDZ ligand, which is responsible for binding to PSD-95 and its MAGUK family members. 

PSD-95, which is also known as “synapse-associated protein 90 (SAP90)”, is coded by the *DLG4* (discs large homolog 4) gene and is one of the most abundant proteins in the upper layer of the complex architecture of the three-layered postsynaptic density (PSD) of the excitatory synapse [34,35]. Mutations of *DLG4*, including sequence variants and copy number variants, have been identified in patients diagnosed with schizophrenia, autism, and intellectual disability [35]. (PSD95/SAP90) is the most studied of the DLG family of top layer scaffolding proteins [35]. Members of the DLG family share the common architectural motif of three N-terminal PDZ domains, an SH3 “src homology domain” and a GUK domain toward the C-terminus of the protein [34]. These domains participate in PSD-95 binding to a large number of interacting partners in the postsynaptic side of the excitatory synapse [34]. The first two of the PDZ domains from the N-terminus of Dlg4 interact with C-terminal PDZ ligands of GluN2A and GluN2B NMDA receptor subunits and the third PDZ domain interacts with neuroligins. Additional Dlg4 interactors include AMPA glutamate receptors, potassium channels, and cytoskeletal proteins. Specifically, some of the PSD-95 direct interacting partners and, in some instances, additional indirectly interacting partners (and the PSD-95 domains responsible for the interaction) include: GluN2 NMDA receptor subunits (PDZ domains 1,2); Stargazin, which itself interacts with GluR1, GluR2, and GluR4 AMPA receptor subunits (PDZ domain 1); K^+^ channel (PDZ domains 1,2); Neuroligin (PDZ domain 3); GKAP, which is responsible for indirect interactions with metabotropic mGluR1/5 receptors and F-actin via SHANK-HOMER and SHANK, respectively (GUK domain); and Arc (PDZ domains 1,2); among other interacting partners [35].

The DLGAP family of scaffolding proteins forms the middle layer and they possess a PDZ ligand motif that binds to the PDZ domain of Shank proteins, which comprise the bottom layer [34]. Moreover, DLGAP proteins interact directly with the dynein light chain when this motor protein is itself associated with the complex of Dlg4 bound to myosin-V [34]. This complex of ‘DLGAP-dynein light chain-Dlg4-myosin-V’ bound and interacting with each other is not only involved in connecting top and bottom layers of this bidirectional macromolecular signaling complex, but suggests that it may also have a role in regulating the transport of DLGAP and DLG proteins to their correct positions within this three layered structure [34]. Importantly, genes for several of the proteins in this complex interaction network of proteins linked to PSD-95 located in the postsynaptic side of the excitatory synapse on dendritic spines are “high-risk ASD genes” (e.g., coding for SHANK, HOMER, neuroligins, and fragile X mental retardation protein 1 (FMR1)). The bottom layer of Shank proteins have regulatory roles in cytoskeletal dynamics and glutamate receptor activity. The Shank proteins interact with cortactin and the Homer family of adaptor proteins, the latter, in turn, couples Shank proteins to metabotropic glutamate receptors (mGluRs). The elucidation of the architecture of the postsynaptic interactome highlights cross-talk between signaling pathways and the complex simultaneous regulation of multiple signaling pathways at the level of the single excitatory synapse. Genetic variants have been identified in coding regions for proteins assigned to all three layers of ‘core scaffolding PSD interactomes’ in persons with ASD, developmental disorders, and intellectual disability [34].

Of particular relevance to the current Review, in addition to its critical roles in NMDA subtype-selective signaling, many of which are unrelated to the ionotropic functions of the NMDA receptor [13], PSD-95 regulates localization and stabilization of GluN2-subtype-selective NMDA receptors and determines the ratio of density of GluN2B-subtype-containing to GluN2A-subtype-containing NMDA receptors to each other in the postsynaptic excitatory membrane and functional consequences of this ratio for cognitive performance and social behavior [35]. Single channel electrophysiological characteristics of GluN2B-containing and GluN2A-containing NMDA receptors differ from each other: inward Ca^2+^ conductance mediated by GluN2B-containing receptors is greater than GluN2A-containing receptors because GluN2B-containing receptors display slower kinetics and a slower decay time compared to GluN2A-containing receptors. Thus, pathologically increased Ca^2+^ conductance due to an overabundance or increased ratio of density of GluN2B-containing to GluN2A-containing NMDA receptors would be expected to have electrophysiological and metabolic consequences, in addition to increasing the risk for excitotoxicity and neuronal death. Studies examining the consequences of knocking down PSD-95 expression with PSD-95 RNAi and its absent expression in a PSD-95 knockout mouse support its role in increasing hippocampal density of GluN2B-containing receptors [35]. There is an age-related neurodevelopmental switch in relative expression of GluN2B to GluN2A expression with GluN2B expression levels higher in early development and declining into adulthood [15,16,17,18,35]. The developmental pattern of PSD-95 expression suggests that it participates in and regulates the declining relative expression of functional GluN2B-containing NMDA receptors because PSD-95 expression levels increase from early development to adulthood and its higher expression stabilizes in adulthood [35]. The phenotype of the PSD knockout mouse with increased expression of GluN2B-containing NMDA receptors models ASD, displaying increased repetitive grooming (a model of repetitive stereotypic behavior) and decreased vocalization (a model of decreased and impaired social communication).

Increasingly, development of targeted NMDA receptor medication strategies recognize the need to address a possibly pathologically disrupted balance of GluN2B (i.e., increased)- to GluN2A (decreased)-containing NMDA receptor activation in at least some presentations of ASD [13,15]. These strategies include recognition of the theoretical value and advantages of GluN2A subtype-selective positive allosteric modulators (PAMs) to readjust this disrupted balance, especially with respect to PAMs limiting off-target side effects and possessing greater spatial and temporal selectivity by acting only when and where endogenous glutamate and co-agonists (i.e., glycine and D-serine) are released in response to socio-cognitive challenges [3,5,7,22,30,31,36].

### 2.5. Casein Kinase 2 (CK2)

Casein Kinase 2 (CK2) is responsible for phosphorylating the serine amino acid at position 1480 in the PDZ ligand, which is the mechanism for removing GluN2B-containing NMDA receptors via lateral movement from the synapse to extrasynaptic sites and eventual endocytosis. With development and neural activity, CK2-mediated phosphorylation of S1480 promotes removal and replacement of GluN2B-containing NMDA receptors with GluN2A-containing NMDA receptors [24]. Upstream regions of the GluN2B C-terminus also have functional and regulatory roles; for example, a CaMKII-binding site upstream of the PDZ ligand is responsible for recruiting CK2 that phosphorylates S1480, which begins the process of removing GluN2B-containing NMDA receptors from synaptic sites by increasing its mobility in the membrane. The C-terminal region also has several secondary non-PDZ binding sites that bind PSD-95 and another MAGUK protein [24].

Very specifically, a recent study revealed that the mouse homologue (S1413L) of the human missense mutation GluN2B S1415L in the C-terminal domain showed impaired binding to MAGUK proteins, had a deficit in its surface expression, and engineered mice expressing the homologous S1413L had fewer dendritic spines [24]. Interestingly, individual channel properties of NMDA receptors constructed with the GluN2B subunit containing this missense mutation were not significantly changed from the wild type receptor, showing that the pathogenic consequences result from reduced trafficking and synaptic targeting of the receptor containing the mutant subunit with a single amino acid substitution in its intracellular C-terminal tail [24]. Again, the data suggest that the major functional deficit associated with this specific missense mutation (S1415L in the human and S1413L in the mouse) results from reduced density of this functional ligand-gated ion channel at synaptic sites, its contribution to reducing the density of synaptic spines, and probable disruption of the three-layered architecture of the postsynaptic density as a result of impaired binding with interacting binding partners [24].

### 2.6. Eps8

“Epidermal growth factor receptor pathway substrate 8 (Eps8)” is an actin cap binding protein that regulates actin polymerization and through this mechanism is involved in regulating size, shape and density of dendritic spines [37]. Eps8 is a multifunctional actin-binding protein that interacts with binding partners (e.g., through its SH3 domain) and, thereby, participates in cell signal transduction pathways, including transduction of signals from Ras to Rac [37]. Importantly, filamentous actin (F-actin) depolymerization increases the mobility/diffusion of AMPA and NMDA receptors between synaptic and extrasynaptic sites, and decreases the “dwell” time of these receptors within the synapse. The polymerization and depolymerization of F-actin, a major cytoskeletal structure enriched in the postsynaptic density of the dendritic spine, is a mechanism of remodeling the spine, whose morphological transformations reflect changes in the functional architecture of the postsynaptic density due to changes in alignment and binding of scaffolding proteins and anchoring of receptors in the synapse, especially ionotropic and metabotropic glutamate receptors [37]. In addition to changes in the polymerization and depolymerization of F-actin influencing membrane diffusion, anchoring and sensitivity to activation of NMDA receptors, NMDA receptor activation itself affects protein interactions between scaffolding proteins and C-terminal tails of GluN1 and GluN2 subunits, which lead to changes in the polymerization state of F-actin. As an actin-capping protein regulating spine remodeling, Eps8 binds to the “barbed” end of the actin filament, which blocks both addition and loss of actin filaments. The actin-capping function of Eps8 is regulated by its phosphorylation. An increased density of immature spines and correlated impairments of learning and memory were found in Eps8 knockout (KO) mice lacking expression of Eps8, which highlighted the functional relevance of Eps8′s role in actin-capping and spine remodeling. Further, lowered expression of Eps8 levels in brain have been reported in ASD.

The relative synaptic expression of hippocampal GluN2B and GluN2A subunits to each other was changed in the Eps8 KO mouse, which supports a regulatory role of actin polymerization/depolymerization in both ionotropic and metabotropic functions of NMDA receptor activation [37]. Whole cell voltage-clamp recordings were conducted in primary cultures of E18 mouse hippocampal neurons derived from Eps8 KO and wild type mice. A lower frequency of NMDA-mediated miniature excitatory postsynaptic currents (mEPSCs) were recorded from the cultures of primary hippocampal Eps8 KO neurons, compared to the cultured wild type neurons. Similarly, NMDA evoked currents were lower in cultured Eps8 KO neurons than wild type neurons, and within the cultured Eps8 KO neurons the ratio of the evoked AMPA current to evoked NMDA current was increased, whereas the absolute increase in the peak amplitude of the evoked AMPA current was unchanged between the Eps8 KO and wild type cultures. Although the frequency of NMDA receptor-mediated mEPSCs recorded in the cultured Eps8 KO neurons was lower, the amplitudes and decay times were increased. These data suggest that although spontaneous NMDA receptor-mediated activity was lower in the Eps8 KO cultured neurons, the absence of Eps8 actin-capping during development led to expression of receptors with different properties, which could reflect differences in subunit compositions and/or post-translational modifications of the subunits. Studies of the effect of ifenprodil (3 µM), a GluN2B subunit-specific antagonist used at a concentration that does not affect GluN2A subunit-containing NMDA receptors, revealed that the changes in amplitude and decay times were in fact related to a relative increase in synaptic expression of GluN2B subunit-containing receptors to GluN2A subunit-containing receptors in the Eps8 KO hippocampal cultures, compared to the wild type cultures [37]. Moreover, when a Zn^2+^ chelator was exposed to the cultures of hippocampal Eps8 KO and wild type neurons, the NMDA receptor-mediated current response was enhanced to a significantly greater extent in the wild type cultures [37]. Zn^2+^ binds to a site on the GluN2A subunit and tonically inhibits GluN2A-containing receptors. Thus, the reduction in Zn^2+^ as a result of its chelation is expected to have a greater effect on cells expressing a greater proportion of GluN2A-containing receptors on their surface in response to the fast local application of a saturating concentration of NMDA, which appears to be the case for the cultured wild type neurons.

An altered ratio of expressed GluN2B to GluN2A subunits incorporated into heterotetrameric NMDA receptors on the surface of cultured Eps8 KO hippocampal neurons relative to the wild type cultures, prompted exploration of whether this altered ratio could be due to cytoskeletal disruption resulting from the absence of the actin-capping function of Eps8 [37]. To address this question, primary cultured hippocampal neurons were exposed to LatB (300 mM from 10 DIV to 14 DIV), which depolymerizes actin and uncouples its association with NMDA receptors during synapse development. The current response evoked by fast local application of a saturating concentration of NMDA in LatB-treated neurons was more sensitive to inhibition by ifenprodil, the GluN2B blocker [37]. Further, using antibodies directed against extracellular epitopes of the GluN2B subunit, LatB treatment was shown to cause increased expression of GluN2B-containing receptors at the synaptic surface. In summary, the data show that actin depolymerization, an effect associated with genetic deletion of Eps8, results in increased expression of GluN2B-containing receptors at the synaptic surface.

Western blot analysis was used to analyze the GluN2 subunit composition of a subcellular fraction of hippocampal homogenate of adult (5-month-old) Eps8 KO and wild type mice enriched in postsynaptic protein, designated the TIF fraction [37]. Whereas a fraction representing a resuspension of total hippocampal homogenate revealed no differences in NMDA receptor subunit composition between Eps8 KO and wild type tissues, the enriched postsynaptic TIF fraction derived from Eps8 KO hippocampus revealed a significantly higher GluN2B content than the enriched fraction derived from wild type hippocampus. Moreover, within the enriched postsynaptic fraction derived from Eps8 KO tissue, a higher proportion of the population of GluN2B subunits were significantly phosphorylated at a site (Tyr1472) in the C-terminal tail, compared to the population of GluN2B subunits in the enriched wild type fraction. The phosphorylated Tyr1472 site in the C-terminal tail of the GluN2B subunit disrupts the binding of this subunit to the AP-2 clathrin-associated adaptor protein complex responsible for the endocytosis of GluN2B. Thus, a failure of GluN2B endocytosis could account for its increased content within the enriched postsynaptic fraction of Eps8 KO hippocampal homogenate [37]. Additionally, data suggest that there is a relative increase in GluN2B-containing NMDA receptors in extrasynaptic sites, which could reflect diminished anchoring of this receptor subtype and increased membrane diffusion between synaptic and extrasynaptic sites.

Functionally, the loss of the actin-capping function of Eps8 in the Eps8 KO mouse interferes with NMDA receptor-mediated induction of long-term potentiation [38]. Clearly, there is great functional significance associated with normally expected developmental regulation of the switch to an increased ratio of GluN2A-containing NMDA receptors to GluN2B containing receptors; expression of GluN2A subunits increases after birth and their content exceeds that of GluN2B subunits by adulthood. Thus, in neuropsychiatric conditions associated with imbalance in activation mediated by functional GluN2A- (e.g., relatively lower) and GluN2B- (e.g., relatively higher) containing NMDA receptors, there is therapeutic interest in developing subtype selective (e.g., GluN2A) positive allosteric modulators to address at least some of the pathological consequences resulting from imbalance of functional GluN2 receptor subtypes [9,11,12].

### 2.7. IRSp53

Data suggest several functions linked to, and regulated by, NMDA receptor activation are not solely dependent on the ionotropic functions of the receptor, but may relate to effects of activation on membrane fluidity, actin dynamics, and alignment of binding partners and scaffolding proteins located within the postsynaptic density [13,39]. Similarly, in some instances, deficits of electrophysiological and behavioral outputs related to disruption of the architecture of the postsynaptic density at excitatory synapses and altered dendritic spine morphology can be reversed by selective activation of the NMDA receptor. Haploinsufficient expression of functional “insulin receptor substrate p53 (IRSp53)”, a “multi-domain scaffolding and adaptor protein” that is critical for the architecture of the excitatory synapse and mature morphological appearance of dendritic spines, is associated with a phenotypic variety of neuropsychiatric disorders, including ASD [39]. Chromosome 17q25 is the coding region for IRSp53, which is thought to contain 17 exons that can be alternatively spliced to express several “domains” [39]. The IMB (I-BAR) domain binds and deforms PI(4,5)P_2_ and PI(3,4,5)P_3_ phosphoinositide-rich regions of membranes and bundles actin filaments. A CRIB-PR domain binds to the GTP-bound and activated form of Cdc42, a small GTPase that is tethered to the membrane. Another domain, an SH3 domain, can bind actin modulatory proteins, such as WAVE-2, M-WASP, and Eps8, among others. Finally, a C-terminal PDZ-binding motif that interacts with diverse scaffolding proteins containing PDZ domains [39]. Thus, IRSp53 acts at the postsynaptic side of excitatory synapses to influence membrane and actin dynamics and non-ionotropic aspects of NMDA receptor-activated cell signaling that is mediated through alignment of interacting protein partners with C-terminal tails of NMDA receptor subunits [13,39].

Consistent with its critical role in dendritic spine development and synaptogenesis, expression levels of IRSp53 in the postsynaptic density is similar to PSD-95, GKAP, Shank and Homer, which are major scaffolding/adaptor proteins [39]. Interestingly, in a mouse with homozygous deletion of IRSp53 (*IRSp53*^−/−^), hippocampal NMDA receptor-mediated neurotransmission in Schaffer collateral-CA1 pyramidal synapses is enhanced; the enhancement is reflected in NMDA receptor-dependent mEPSCs, evoked EPSCs, and induction of long-term potentiation [39]. The mechanism of this NMDA receptor-mediated enhancement in *IRSp53*^−/−^ hippocampal neurons may be an abnormal stability of actin filaments that resist NMDA receptor activity-dependent removal of NMDA receptors from the synapse. Further, behavioral phenotyping of the *IRSp53*^−/−^ mouse showed impaired social interaction in both the three-chamber apparatus and direct social interaction tests. Male *IRSp53*^−/−^ mice also emitted reduced ultrasonic vocalizations in the presence of a stranger female, consistent with impaired socialization. Thus, normal “socialization” may require an optimal level of hippocampal NMDA receptor activation, too little or too much activation may lead to deficits of sociability, which is similar to the conceptualization of an optimal level of mTORC1 signaling activity for normal socialization [40,41,42,43]. Of interest, there was a dissociation between domains of ASD symptomatology/psychopathology modeled in the *IRSp53*^−/−^ mouse because this mouse model did not show increased repetitive behaviors, such as grooming and digging. The *IRSp53*^−/−^ mouse did show deficits of both spatial learning in the Morris water maze and novel object recognition, but the cognitive deficits showed selectivity as contextual fear conditioning increased when tested 24 h after the training [39].

The increased level of hippocampal NMDA receptor-activation stimulated interest in exploring possible therapeutic effects of memantine, an uncompetitive antagonist, in the *IRSp53*^−/−^ mouse [39]. The data showed that acute administration of memantine improved social interaction in the three-chamber apparatus and novel object recognition. Moreover, because of the cross-talk between the ionotropic NMDA receptor and metabotropic glutamate receptor 5 (mGluR5) in the excitatory synapse [13], MPEP (2-methyl-6-(phenylethynyl)pyridine), a negative allosteric modulator (NAM) of mGluR5 was tested in the *IRSp53*^−/−^ mouse. MPEP was shown to improve social interaction in this mouse model. Future strategies could consider combinations of memantine and a PAM for GluN2A-containing NMDA receptors; in this manner, it may be theoretically possible to dampen the neurotoxic GluN2B subtype-selective contributions to pathologically elevated hippocampal NMDA receptor activation, while preserving or increasing the positive therapeutic effects of GluN2A-containing NMDA receptors on sociability and cognition in the *IRSp53*^−/−^ mouse model. Additionally, given presentations of ASD linked to both NMDA receptor hypo- and hyperfunction, it will be important in this era of Personalized Medicine to genotype persons presenting with clinical manifestations of ASD. Ideally, the earlier genetic “diagnoses” can be made to allow for earlier consideration of disease-modifying medication interventions that address known etiologies and mechanisms of pathogenesis [4,29,44]. Of course, earlier diagnosis will lead to earlier interventions implemented in all of the relevant components of an individualized, multimodal, interdisciplinary treatment plan, such as peer socialization, special education, and speech and language therapy, among other necessary components.

### 2.8. TBR1

Haploinsufficient expression of functional T-box brain 1 (TBR1), a neuron-specific transcription factor expressed in cerebral cortex, hippocampus, amygdala and olfactory bulb, is associated with ASD [44]; most commonly, recurrent heterozygous de novo mutations of *TBR1* are discovered in these cases of sporadic ASD via whole exome sequencing. Absent expression of *Tbr1* in the homozygous *Tbr1*^−/−^ mouse is associated with neonatal lethality and disrupted forebrain development, including defects of cortical axonal projections and neuronal migration to the cerebral cortex and amygdala. In order to better understand the pathogenesis of ASD associated with heterozygous de novo mutations in only one of the parental *TBR1* alleles, anatomic, histopathological, gene expression and behavioral studies were conducted in the heterozygous *Tbr1*^+/−^ mouse to determine effects of haploinsufficient expression of *Tbr1* [44]. Of particular interest to the current Review, D-cycloserine, an NMDA partial agonist, was able to attenuate behavioral deficits in the *Tbr1*^+/−^ mouse [44].

Whereas effects on lamination of the cerebral cortex, consistent with impaired neuronal migration seen in the homozygous *Tbr1^−/−^* mouse, were not observed in the haploinsufficient *Tbr1*^+/−^ mouse, the latter mouse is missing the posterior part of the anterior commissure, which connects the two amygdalae of the two hemispheres [44]. Inter-amygdalar disconnection was reflected in absent or reduced labeling of the posterior part of the anterior commissure and amygdala contralateral to the sites of implantation seen with retrograde tract-tracing labeling when a lipophilic dye and fluorescent signals of red beads were implanted into basolateral and lateral amygdala, respectively, of the *Tbr1*^+/−^ mouse. Moreover, when the red beads were implanted into the central amygdala, intra-amygdalar disruptions of ipsilateral connections between the central amygdala and basolateral amygdala were seen. Thus, haploinsufficiency of *Tbr1* affects expression of genes involved in regulating amygdalar axonal projections [44]. 

Microarray analysis of embryonic forebrains followed by quantitative PCR using amygdalar RNA were used to explore downstream effects of *Tbr1* haploinsufficiency on genes that may affect amygdalar axonal projections [44]. Altered expression of three genes (i.e., *Ntng1*, *Cdh8*, and *Cntn2*) that control neuritic outgrowth and fasciculation were expressed in embryonic forebrain of *Tbr1*^+/−^ mice and also expressed in amygdala were of interest. Specifically, in both adult and one-day-old amygdalae, there was upregulation of *Ntng1* and *Cdh8* expression and downregulation of *Cntn2* expression in the *Tbr1*^+/−^ mouse [44]. When amygdala neurons from wild type and *Tbr1*^+/−^ mice were cultured, the axonal phenotypes of the cultured *Tbr1*^+/−^ amygdalar neurons at 4, 7, and 10 days in vitro (DIV) were shorter and abnormal (i.e., multiple axons developed). Further, whereas overexpression of wild type *Tbr1* rescued the axonal phenotype of the cultured *Tbr1*^+/−^ amygdalar neurons, expression of a mutant transcript identified in association with ASD did not rescue the axonal phenotype of cultured *Tbr1*^+/−^ amygdalar neurons [44]. Manipulation of expression of individual target genes of *Tbr1* (i.e., knockdown of *Cdh8* and *Ntng1* and overexpression of *Cntn2*) rescued the axonal phenotype of cultured *Tbr1*^+/−^ amygdalar neurons (i.e., their reduced primary axonal length and higher percentage of multipolar neurons). However, the functional contribution of each manipulated target gene to normalized axonal outgrowth in the cultured *Tbr1*^+/−^ amygdalar neurons was thought to be overlapping and not identical [44].

These findings regarding the ability of transcripts that knockdown expression of Ntng1 and Cdh8 (i.e., miR-Ntng1 and miR-Cdh8) and promote expression of *Cntn2* in *Tbr1*^+/−^ amygdalar neurons cultured in vitro were “predictive” of similar effects of these transcripts on axonal outgrowth in vivo. In utero electroporation (IUE) was used to transfect these transcripts into amygdala of *Tbr1*^+/−^ embryos, axons extended to the anterior commissure and crossed the midline, and the morphology of the extended amygdalar processes appeared “more similar to that of the wild type neurons” [44]. Interestingly, the morphology of cortical and striatal neurons was described as “comparable” between *Tbr1*^+/−^ and wild type mice [44]. Thus, morphological effects of *Tbr1* and the three downstream target genes of this transcription factor (i.e., *Ntng1*, *Cdh8*, and *Cntn2*) appear to have selectivity for differentiation of amygdalar neurons. Of interest, *Tbr1* regulates expression of *Reln*, a gene involved in neuronal migration and corticogenesis, and *Grin2b*, a gene coding an NMDA receptor subunit [44].

*Tbr1*^+/−^ mice show a variety of social and cognitive deficits supporting their modeling of neurodevelopmental disorders, including ASD [44]. Further, the data support roles for the amygdala in socialization, beyond a known role in fear conditioning and aversion of fearful faces. *Tbr1*^+/−^ mice showed deficits of conditioned taste aversion and auditory fear conditioning. Compared to the wild type controls, the *Tbr1*^+/−^ mice showed deficits of reversal learning in an appetitive-motivated T-maze test and two-choice digging test, perhaps signifying a loss of cognitive flexibility and a tendency to perseverate in their performance of previously learned responses when conditions for receiving a food reward changed. *Tbr1*^+/−^ mice showed deficits of sociability in the three-chamber apparatus; reduced reciprocal social interactions; defective social transmission of food preference; and *Tbr1*^+/−^ mouse pups isolated from their mothers emitted fewer ultrasonic vocalizations; compared to wild type controls [44].

The impaired inter- and intra-amygdalar connectivity of *Tbr1*^+/−^ mice was reflected in decreased number of c-Fos-positive cells/mm^2^ in the lateral amygdala after both conditioned taste aversion and auditory fear conditioning, and a decreased number of c-Fos-positive cells/mm^2^ in the basal amygdala after conditioned taste aversion [44]. Activation of c-Fos-positive cells in hippocampal CA1, CA2, and CA3 regions did not differ between *Tbr1*^+/−^ and wild type mice after conditioned taste aversion and auditory fear conditioning. The activated c-Fos-positive cells were mainly those expressing *Tbr1*.

Grin2b protein expression increased in the lateral amygdala of wild type mice after conditioned taste aversion with no increase in Grin2b expression in the *Tbr1*^+/−^ mice [44]. Moreover, there was higher Grin2b expression in the basal amygdala of wild type mice than that found in the *Tbr1*^+/−^ mice after conditioned taste aversion. Importantly, wild type and *Tbr1*^+/−^ mice did not differ in their expression levels of Grin2a and Grin1 after conditioned taste aversion. Unlike the wild type mice where conditioned taste aversion was associated with upregulated expression of *Ntng1* and *Cdh8*, and downregulated expression of *Cntn2* in the amygdala, there was no altered expression of these genes after conditioned taste aversion in the *Tbr1*^+/−^ mice [44].

Importantly, NMDA receptor activation in the amygdala of *Tbr1*^+/−^ mice by bilateral infusion of D-cycloserine into the basolateral amygdala attenuated deficits of reciprocal social interaction and several cognitive deficits emerging after conditioned taste aversion [44]. Similarly, deficits of reciprocal social interaction and cognitive deficits related to conditioned taste aversion could be created by the infusion of ifenprodil, a Grin2b antagonist, into the amygdalae of wild type mice. Intraperitoneal injection of D-cycloserine was also effective in improving reciprocal social interaction and cognitive deficits of the *Tbr1*^+/−^ mice [44]. Overall, these data strongly support a therapeutic role for selective activation of Grin2b-containing NMDA receptors in the *Tbr1*^+/−^ mouse model, a model that has deficits in upregulation of its expression of the Grin2b NMDA receptor subunit. In this era of personalized medicine, these data have translational implications supporting genotyping of persons with ASD, a heterogeneous group of highly heritable neurodevelopmental disorders, whose treatments may differ with respect to selective activation and selective dampening of NMDA subtype-selective receptors.

### 2.9. TCF4

Overexpression of Transcription Factor 4 (TCF4), a basic helix–loop–helix transcription (bHLH) factor, occurs naturally as a result of microduplications on chromosome 18 in the region containing the gene for TCF4 [45]. Microduplications, microdeletions, and sequence variants, including single nucleotide polymorphisms within introns of TCF4, are associated with variable phenotypic expression of neurodevelopmental disorders, such as schizophrenia, autism and intellectual disability. Clinical data suggest that both gain and loss of TCF4 function can adversely affect cortical developmental and serve as a first-hit in the pathogenesis of neuropsychiatric disorders (e.g., via increased gene dosage of TCF4 secondary to microduplications, reduced gene dosage of TCF4 secondary to microdeletions, or reduced transcriptional efficiency secondary to mutations affecting binding to DNA and/or causing decreased binding of TCF4 with interacting protein partners involved in transcriptional activation, respectively).

Binding to specific motifs in the DNA requires homodimerization of TCF4 or its heterodimerization with other bHLH transcription factors, and the ability of TCF4 to promote transcription is inhibited by calmodulin in a Ca^2+^-dependent manner, consistent with possible activity-dependent regulation of its transcriptional efficiency [45]. In utero electroporation (IUE) performed on embryonic day 16 (E16), a timepoint just prior to neurogenesis, was used to deliver a *TCF4* construct capable of promoting regionally selective expression of gain-of-function TCF4 protein in medial prefrontal cortex (mPFC) and somatosensory cortex (SCx) of developing rat embryos. Expression of gain-of-function TCF4 in utero disrupted the columnar distribution of layer 2/3 pyramidal neurons selectively in mPFC, whereas it was without similar effect in the SCx [45]. The formation of compact cellular aggregates of prefrontal pyramidal neurons selectively in mPFC of developing rat embryos with electroporation of a gain-of-function *TCF4* construct in mPFC suggests that an effect of TCF4 overexpression includes regionally selective impairment of migration of newly differentiated neurons to the cortex [45]. Specifically, electroporation of a full-length functional isoform of *TCF4* (i.e., *TCF4B*) causing *TCF4* “gain-of-function” disrupted the distribution of pyramidal neurons with formation of compact cellular aggregates apparent on postnatal day 1 (PND 1). The disruption of the columnar distribution of prefrontal pyramidal cell neurons was not seen with a “loss-of-function” construct, a construct containing a point mutation in the bHLH domain that renders it unable to bind to DNA, or a construct lacking expression of the AD2 domain, which deletes the protein interaction domain that binds necessary mediators of transcriptional activation to TCF4 [45]. Ca^2+^-dependent inhibitory regulation of TCF4-activation was shown by co-expression of calmodulin with TCF4B, the full-length TCF4 isoform whose expression is associated with “gain-of-function”; pyramidal cell aggregation was not observed in the context of co-expression of TCF4B and calmodulin, which was shown to be dependent on the binding of Ca^2+^ by calmodulin. When neuronal excitability was reduced via co-expression of a recombinant inward rectifying potassium ion channel (Kir2.1) with TCF4B, the disruptive effect of “gain-of-function” TCF4 on columnar organization was prevented [45]. Thus, neuronal activity is necessary for the disruptive effect of TCF4 overexpression on pyramidal cell distribution and columnar organization to be observed [45]. 

Functional deletion of *Grin1*, the gene encoding GluN1, using CRISPR/Cas9 was used to show that NMDA receptor-mediated Ca^2+^ conductance contributes to the activity-dependent regulation of TCF4-directed transcription. Specifically, cellular aggregation and abnormal columnar formation in mPFC was not observed when TCF4B was co-expressed with CRISPR/Cas9 suppression of NMDA receptor-function [45]. Moreover, data suggested that TCF4 gain-of-function actually leads to a greater amount of NMDA receptor activity, which in early development might lead to pathologically increased expression of GluN2B-containing NMDA receptors [45]. A temporal context may be necessary for understanding effects of NMDA receptor activation on TCF4-transcriptional activation since NMDA receptor-mediated Ca^2+^-dependent regulatory influences on TCF4-transcriptional activation emerge when neurons reach the cortical plate.

Overall, these data support a refinement of thinking about the role of abnormalities of NMDA receptor-mediated neurotransmission and their possible etiopathogenic contributions to neurodevelopmental disorders. NMDA receptor-mediated neurotransmission must be considered and understood in the context of developmental stage, anatomic region (e.g., mPFC versus SCx), and ratio of expressed GluN2B- to GluN2A-subunit-containing NMDA receptors in synaptic versus extrasynaptic sites, among other considerations. Of relevance to the current Review, NMDA receptor-mediated Ca^2+^ conductance appears to influence transcriptional consequences of overexpressed or gain-of-function TCF4 constructs. As noted, the temporal activation of NMDA receptor-mediated Ca^2+^ conductance is reported to coincide with migrating neurons reaching the cortical plate and ceasing their migration [45]. These NMDA receptor-mediated Ca^2+^ currents are necessary for dendritic growth and synaptogenesis. Thus, NMDA receptor-mediated Ca^2+^ currents were hypothesized to play a significant role in activity-dependent regulation of TCF4-directed transcription [45]. Given the developmental regulation of GluN2B- and GluN2A-containing NMDA receptors, the data suggest that pathogenic effects of early disruption of GluN2B-containing NMDA receptor-mediated activation, whose expression predominates over GluN2A-containing NMDA receptors during early development, may itself result, at least in part, from subtle and selective disturbance of the columnar distribution of layer 2/3 pyramidal neurons in prefrontal cortex [15,16,17,18,46]. In any event, there is much interest in a growing list of mutations of GluN2B and GluN2A receptor subunits themselves that directly alter NMDA receptor function and are associated with variable expression of neuropsychiatric disorders, such as intellectual disability, autism and epilepsy [47].

### 2.10. Regulation of GluN2B via Endosomal Recycling and KIF21B

Endosomal recycling is a mechanism for regulating the density of GluN2B-containing NMDA receptors at the surface of the cell that has functional consequences with respect to mouse social behavior [48]. Recycled and internalized endosomes containing GluN2B-NMDA receptors can undergo lysosomal degradation or serve as a pool of reserve receptors for reinsertion into the synapse. Neurobeachin (NBEA), a 37 kDa multidomain protein, and KIF21B work cooperatively to assure that recycled endosomes enclosing GluN2B-containing NMDA receptors are targeted to the synapse and, then, “unloaded” when they arrive, respectively. KIF21B is recruited to the endosomal trafficking machinery where it complexes with Rab4 GTPase. Kinesin and dynein are the motor effectors of Rab4 activation, and they are responsible for cargo transport, including transport of recycled endosomes, along the microtubule skeleton [48]. An activity-dependent destabilization of microtubules is thought to facilitate detachment and unloading of trafficked cargo. KIF21B causes microtubule “catastrophes” and pausing and, thereby, plays a complex role in microtubule remodeling and unloading of endosomes targeted to the synapse. Thus, a lack of functional KIF21B would be predicted to impair the delivery of GluN2B-continaing NMDA receptors to the cell surface. Knockout of both Nbea expression (*Nbea*^−/−^ mice) and Kif21b expression (*Kif21b*^−/−^ mice) in mice was associated with reduced GluN2B expression on the surface of the neuron [48]. A failure to unload its cargo of recycled GluN2B-NMDA receptors contained within trafficked endosomes may explain the reduction in the surface density of GluN2B-containg NMDA receptors in *Kif21b*^−/−^ knockout mouse brains [48]. This loss of unloading (i.e., targeted detachment) in the *Kif21b*^−/−^ mouse may be due to increased microtubule stability. Again, NBEA and KIF21B functionally interact and associate with Rab4 GTPase and other proteins constituting and regulating the endosomal trafficking machinery (via altering microtubule dynamics and effects on kinesin and dynein motor proteins). The endosomal trafficking machinery acts to transport, recycle, target, and unload endosomes enclosing GluN2B-containing NMDA receptors at the excitatory synapse.

Whereas activity levels (i.e., distance moved) and preference for an enclosed novel stimulus mouse over an inanimate object did not differ between *Kif21b*^−/−^ and wild type mouse strains in the three-chamber mouse sociability apparatus, the *Kif21b*^−/−^ mouse differed from the wild type mouse by its lack of significant preference for exploring a novel stimulus mouse over a familiar mouse, consistent with impaired social recognition memory [48]. Moreover, when the actual total amounts of time *Kif21b*^−/−^ and wild type mice spent exploring the stimulus mouse versus an inanimate object in the social preference paradigm and the “stranger” mouse versus the familiar mouse in the social recognition paradigm were calculated, lack of expression of Kif21b was associated with significantly reduced total amounts of time. These data support involvement of Kif21b in the initiation of social approach and social cognition via its effect on reducing the synaptic density of GluN2B-containing NMDA receptors [48].

Variants of GRIN2B were associated with autism in a Chinese population, including variants coding for the ion selectivity region of the channel and the carboxy-terminal domain (CTD), the latter domain interacts with binding partners (e.g., CAMKII, PSD-95 and α-Actinin) [32]. Further, a de novo breakpoint in the neurobeachin (NBEA) gene on chromosome 13q was identified in a male patient with autism. Thus, variants of GRIN2B and NBEA confer risk to autism, supporting therapeutic strategies to modulate endogenous.

NMDA receptor-mediated transmission in ASD. Increasingly, optimal sociability and cognitive performance are thought to reflect a finely tuned balance between activation of GluN2B and GluN2A-containing NMDA receptors to each other that will require adjustment for developmental age, anatomic location, and cell type, in addition to the demands of specific socio-cognitive tasks [13,15].

**Table 1:** Summary of the subunits, modulators, and positive allosteric modulators (PAMs) of N-methyl- D-aspartate receptors (NMDARs).

		Function	Human Chromosomal Location	Species	Relationship to NMDA Receptor	Brain Area	Ref
*16p11.2 locus*		Hotspot for CNVs	16p11.2	Mouse	This deletion of 16p11.2 was seen in people with ASD and led to behavioral deficits; restoring normal NMDAR function through chemogenetic manipulation led to improvements of the behavioral deficits associated with ASD.	Frontal cortex	[25,26]
*NMDAR* Subunits	*GRIN1*	Integral to NMDA function	9q34.3	Mouse	GRIN1 is reduced in ASD patients; splice variants regulated sensitivity of NMDAR; exon 5 isoforms influenced modulators of NMDAR.	Hippocampus	[1,49]
	*GRIN2A*	Improved NMDA receptor mediated long-term potentiation (LTP) induction compared to GluN2B	16p13.2	Mouse	PAMs were a possible avenue for the pharmacotherapeutics of Alzheimer’s Disease and ASD; NMDAR (with GluN2A subunits) was a possible treatment method for ASD.	Cortical regions	[12]
	*GRIN2B*	Codes for GluN2B; phosphorylated the serine amino acid at position 1480 in the PDZ ligand	12p13.1	Mouse Rat	Splice variants were prevalent in ASD patients; people with a S1413L variant of GluN2B (on the NMDAR) showed decreased functional deficit because of the reduced density of the channel, not the channel itself.	Hippocampus	[24,50]
NMDAR Modulators	*Cul3*	Ubiquitination; proteasomal degradation; cytoskeletal organization; cell differentiation	2q36.2	Mouse	Impaired sociability; Cul3 deficiency caused NMDA hypofunction, which led to impaired sociability.	Prefrontal cortex	[27]
	*DLG4* (PSD95)	Scaffolding protein; Recruited NMDA and K^+^ channel cluster	17p13.1	Mouse	Patients with ASD had genetics variants in proteins of all three layers of ‘core scaffolding PSD interactomes; *DLG4* produced various splice variants of NMDARs.	Cortex	[34]
NMDAR Modulators	*Casein* *Kinase 2*	Phosphorylated the serine at position 1480 in the PDZ ligand	20p13	Mouse Rat	Mice with S1413L variants had fewer dendritic spines. This lack was due to reduced trafficking, which was normally regulated by CK2, but could not be since the serine amino acid has been substituted.	Hippocampus Cortex	[24]
	*Eps8*	Actin cap binding protein; regulated actin polymerization and dendritic spine shape and density	12p12.3	Mouse	Eps8 levels were lower in the brains of ASD patients compared to controls; without Eps8, NMDARs produced fewer micro excitatory post synaptic circuits (mEPSCs).	Hippocampus	[37,38]
	IRSp53	Critical for the architecture of the excitatory synapse and mature morphological appearance of dendritic spines	17q25	Mouse	*IRSp53*^−/−^ mice showed social and cognitive deficits, but not repetitive behaviors; uncompetitive agonist restored social interaction.	Hippocampus	[39]
	*TBR1*	Regulated expression of *Reln* (involved in neural migration and corticogenesis)	2q24.2	Mouse	*Tbr1*^+/−^ mice displayed deficits in sociability and social interaction; activating NMDA receptors was able to attenuate deficits in reciprocal social interaction.	Amygdala	[44]
	*TCF4*	Promotes transcription	18q21.2	Rat	Variants of *TCF4* produced variable phenotypic expression of ASD; NMDAR regulated Ca^2+^ conductance regulated TCF4-directed transcription.	Medial prefrontal cortex	[45,47]
	*Kif21b*	Endosomal recycling	1q32.1	Mouse	Variation of the Grin2B (which is trafficked by *KIF21B*) in *Kif21b^−/−^* Mice was associated with ASD; reduced GluN2B expression on the surface of the neuron.	Hippocampus	[32,48,51]

## 3. p-Cresol, a Pharmacological Tool to Disturb the Balance between GluN2B and GluN2A NMDA Receptor Subunits

Gastrointestinal complaints occur commonly in persons with ASD, arousing interest in possible pathogenic consequences of abnormalities of “tight junctions” between intestinal epithelial cells leading to disruption of the gut-blood barrier, as well as alterations of the gut microbiota [52,53,54,55]. With respect to the gut microbiota, a recent study suggested that p-cresol, a neurotoxin produced by several species of *Clostridium* that differentially alters relative expression of GluN2B and GluN2A NMDA receptor subunits to each other in hippocampus and nucleus accumbens of “control” Wistar and audiogenic seizure-prone Krushinski–Molodkina (KM) rats [52]. p-Cresol binds covalently to dopamine β-hydroxylase and, thereby, may increase relative concentrations of dopamine via interference with the production of norepinephrine [52]. Interestingly, p-cresol is reported to impair sociability in the rat and lower the seizure threshold in seizure-prone rats, which may be related to p-cresol alterations of expression and ratios of GluN2 receptor subtypes to each other. The data have implications for possible influences of dopaminergic transmission on NMDA subtype-selective activation and changes in downstream signaling pathways involving CREB (cyclic-AMP response element binding protein) and Rac1, a member of the Rho family of GTPases that is involved in regulating actin dynamics and dendritic spine formation [52,56].

In experiments exploring effects of p-cresol on GluN2 subtype-selective expression [52], groups of Wistar rats and audiogenic seizure-prone KM rats received daily intraperitoneal injections of p-cresol (30 mg/kg) or saline for 21 days and sacrificed three days after cessation of injections. Following decapitation, studies were conducted in extracted hippocampus and nucleus accumbens. Treatment with p-cresol increased GluN2A protein content in hippocampus of comparator Wistar rats and nucleus accumbens of the seizure-prone KM rats. Treatment with p-cresol did not significantly affect GluN2B protein content in hippocampi of either rat strain. However, p-cresol had opposite effects on the GluN2B content in nucleus accumbens of comparator Wistar and seizure-prone KM rats: increasing the GluN2B content in the Wistar strain and decreasing the GluN2B content in the seizure-prone KM strain [52]. Moreover, p-cresol caused significant changes in the ratios of GluN2B to GluN2A protein content as a function of interaction between treatment and strain. Thus, the GluN2B/GluN2A ratio was increased in the nucleus accumbens and decreased in the hippocampus of the Wistar strain, but the GluN2B/GluN2A ratio was decreased in the nucleus accumbens and increased in the hippocampus of the seizure-prone KM strain. Ordinarily, distribution of GluN2B-containing NMDA receptors is enriched in extrasynaptic sites relative to synaptic sites and their selective activation induces mitochondrial dysfunction and promotion of cell death [52]. Thus, there was interest in exploring effects of p-cresol-induced changes in the GluN2B/GluN2A ratio on downstream signaling intermediates. Changes in the protein content of GluN2 receptor subtypes and altered GluN2B/GluN2A ratios as a function of p-cresol treatment and anatomic region influenced levels of Ser133-phosphorylated-CREB in the nuclear fractions of nucleus accumbens and hippocampus, suggesting a negative correlation between CREB activation and GluN2B protein content. Thus, p-cresol was associated with decreased content of phosphorylated (activated)-CREB in nucleus accumbens of Wistar rats and decreased content of phosphorylated-CREB in hippocampus of seizure-prone KM rats [52]. The effect of p-cresol treatment on the Rac1 content in the “cytosol fractions” of nucleus accumbens and hippocampus, a measure of Rac1 activation, showed complex interactions between region and treatment. Thus, p-cresol treatment lowered Rac1 activation in nucleus accumbens of both the Wistar and seizure-prone KM rats, and increased Rac1 activation in hippocampus of both rat strains [52].

Alterations of relative expression of GluN2 receptor subunits to each other in hippocampus with functional implications for sociability and cognition have been described in rodent models [57,58]. These current data suggest that p-cresol, a gut-derived neurotoxin associated with *Clostridium* species, might independently contribute to altered ratios of GluN2B/GluN2A with functional behavioral consequences. Moreover, p-cresol-induced alterations of dopaminergic tone in the nucleus accumbens, which receives dense projections from midbrain dopaminergic nuclei, and alterations of NMDA receptor subtype-selective activation within this anatomic site may interact to affect rewarded behaviors and motivation. Additionally, the data suggest that regionally selective alterations of GluN2B/GluN2A ratios may be associated with changes in downstream signaling pathways. Thus, p-cresol production and leakage across the gut–blood barrier could conceivably be relevant to phenotypic manifestations of ASD and selection of pharmacotherapeutic strategies.

## 4. Presence or Absence of GRIN1 Exon 5 Isoforms Influences the Allosteric Modulatory Properties of Zn^2+^, Mg^2+^, and Spermine on NMDA Receptor Function

Ordinarily, *GRIN1* mRNA transcripts are alternatively spliced leading to a mixture of isoforms, some of which contain exon 5 that codes for an N1 cassette of a 21-amino acid sequence that is highly conserved across vertebrate species, and some lack exon 5 [49]. In rat hippocampus, about 38% of the *Grin1* transcripts contain exon 5; GluN1b refers to the translated receptor subunit of the *Grin1* mRNA transcript containing exon 5, whereas GluN1a is the translated receptor subunit lacking this N1 cassette. When translated, the sequence of 21 amino acids contained within the N1 cassette lies in a region of the GluN1b amino terminal domain (NTD) that interfaces with the ligand binding domains (LBDs) of both the GluN1b and GluN2 receptor subunits. Importantly, absence of the N1 cassette does not influence assembly of functional GluN1a-containing NMDA receptors [49].

A homologous recombination strategy was used to generate homozygous GluN1a mice that lacked expression of the N1 cassette and homozygous GluN1b mice whose translated receptor subunits always contained the N1 cassette. Heterozygous GluN1a mice were bred with each other, as heterozygous GluN1b mice were bred separately with each other, in order to generate the homozygous mice and their wild type littermates [49]. The purpose of creating the homozygous mice that lacked (GluN1a) or possessed (GluN1b) the N1 cassette and wild type littermates that expressed a mixture of GluN1a and GluN1b subunits was to explore the contribution of the N1 cassette to NMDA receptor function. Additionally, the proportion of *GRIN1* transcripts containing exon 5 is reported to be reduced in postmortem cortex obtained from persons with ASD [49].

Presence or absence of the N1 cassette did not affect the overall expression of total *Grin1* mRNA nor did its absence or presence influence the expression of GluN2A and GluN2B NMDA receptor subunits or the expression of the main AMPA receptor subunits in the adult CNS [49]. The effects of the absence (GluN1a) and presence (GluN1b) of the N1 cassette on NMDA receptor channel function were studied via conventional whole-cell patch-clamp recordings of primary forebrain cultured neurons prepared from E16 to E17 fetal brains; unless otherwise stated, recordings were made with the voltage clamped at −60 mV [49]. EC_50_ values for concentrations of NMDA and glycine did not differ in primary forebrain cultured neurons prepared from homozygous GluN1a, homozygous GluN1b mice, and wild type mice. Thus, the N1 cassette does not affect the agonist potencies of NMDA and glycine. The absence or presence of the N1 cassette did not influence the IC_50_ values for Mg^2+^ blockade when the membrane potential was clamped and held constant at −60 mV. However, the ability of Zn^2+^, an allosteric inhibitor, to inhibit conductance when the voltage was clamped at −60 mV was lowered in the presence of the N1 cassette. Thus, the IC_50_ value for Zn^2+^ was significantly increased in the presence of the N1 cassette (i.e., in GluN1B neurons), indicating a lowered inhibitory effect compared to wild type neurons [49]. Mg^2+^ has complex effects on NMDA receptor-mediated currents that are dependent on the membrane potential; whereas Mg^2+^ inhibits conductance at negative membrane potentials (e.g., −60 mV), it can act as a positive allosteric modulator, potentiating current flow at positive membrane potentials (e.g., +60 mV). Interestingly, the absence or presence of the N1 cassette in the GluN1 subunit was shown to regulate the sensitivity of the NMDA receptor to modulatory effects of Mg^2+^ when the membrane potential was clamped at +60 mV [49]. Specifically, Mg^2+^ (10 mM) potentiated current flow when the N1 cassette was absent (GluN1a) in GluN1 subunits and decreased current flow when the N1 cassette was present (GluN1b). The positive allosteric modulatory effects of spermine (500 µM), a naturally occurring polyamine, on NMDA receptor-mediated current flow at negative membrane potentials was also shown to be regulated by the absence or presence of the N1 cassette in the GluN1 subunit [49]. Spermine increased current amplitude in forebrain neuronal cultures derived from GluN1a and wild type mice, but reduced current amplitudes in cultures derived from GluN1b neurons. Further, the modulatory effects of spermine on NMDA receptor-mediated ion conductance were complex, decreasing the decay time of these currents in GluN1a and wild type neurons, but increasing the decay time in GluN1b neurons. Thus, the absence or presence of the N1 cassette in the fully assembled, functional heterotetrameric NMDA receptor influences allosteric modulatory properties of Zn^2+^, Mg^2+^, and spermine [49].

The induction of long-term potentiation (LTP) in the hippocampal CA1 region by theta burst stimulation of Schaffer collateral input was reduced in the presence of the N1 cassette in the hippocampal slices derived from the homozygous GluN1b mice [49]. Moreover, the N1 cassette had functional behavioral significance influencing spatial learning and spatial memory in the Morris water maze. Specifically, homozygous GluN1a mice learned the location of the submerged platform more quickly than the homozygous GluN1b and wild type mice, and also showed better retention of the quadrant in which the submerged platform was located.

As noted, there are postmortem data showing that the proportion of *GRIN1* transcripts containing exon 5 is reduced in persons with ASD. Consistent with this finding, the Mg^2+^ potentiation at a clamped +60 mV membrane potential of induced pluripotent stem cells (iPSCs) generated from dermal fibroblasts of a person with ASD and differentiated into neurons was significantly larger than similar differentiated neuronal cells derived from an unaffected first-degree relative serving as a control [49]. These data are consistent with a reduced proportion of exon 5-containing cassettes in the patient’s iPSC-derived neurons. In any event, the authors speculated that the wild type condition with a mixture of GluN1a and GluN1b subunits is optimal, considering issues pertaining to anatomic distribution, developmental regulation of expression, and unique, but necessary, properties of individual neurons integrated into complex neural circuits. Of relevance to the current Review, these data show that a variety of known and as yet unknown factors influence allosteric modulatory properties of endogenous ligands and cations with possible implications for translational development of PAMs and NAMs as medications for the treatment of ASD. Nonetheless, medication development programs for therapeutic PAMs and NAMs must be encouraged because they offer theoretical advantages over existing “proof of principle/proof of concept” tools that have been tested.

## 5. Anti-NMDA Receptor Antibodies in ASD

In addition to an encephalitis characterized by an array of neuropsychiatric signs and symptoms that can be mistaken for psychosis and catatonia, elevated titers of anti-NMDA receptor antibodies in serum and CSF have been associated with impaired sociability (e.g., lack of empathy and difficulty apprehending intentions of others) [59,60,61]. For example, a normally developing 9-year-old boy was described with a history of good social and academic performance, whose initial presentation was an acute onset of secondary generalized seizures that progressed to an agitated catatonia with posturing and dyskinetic movements, and followed by a “robotic state” with mutism, negativism, and marked social withdrawal [59]. After an extensive diagnostic assessment, anti-NMDA-receptor antibody titers were found to be “slightly raised” in serum and “highly raised” in CSF. Prior to the confirmation of an anti-NMDA-receptor encephalitis, the child had been provisionally diagnosed with “acute late onset autism”. Fortunately, the child recovered over a period of about six months with only the residual of “mild cognitive dysfunction”; his treatment included ECT for the catatonia and rituximab, a monoclonal antibody that targets CD20, a protein expressed on the surface of B-lymphocytes and, thereby, triggers their death [59]. These data are consistent with a possible role for autoimmunity in impaired NMDA receptor-mediated signaling and pathogenesis of impaired sociability observed in at least some patient with ASD. Moreover, greater sensitivity to encephalitic effects of anti-NMDA receptor autoantibodies, such as catatonia, in at least some persons with autism would be consistent with their further exacerbation of already basally impaired NMDA receptor-mediated signaling [60].

Two cases were presented of persons diagnosed with intellectual disability and autism, which are diagnosed in childhood, who were discovered to have anti-NMDA-receptor encephalitis as adults [60]. A 32-year-old single woman diagnosed with mild intellectual disability and autism presented and was treated with antidepressants for what was initially suspected to be depression because of “persistently low mood”, social withdrawal, and sleep and appetite disturbance [60]. Her condition progressively deteriorated to the point that her parents “had to attend to her basic needs” and she manifested hallucinations and marked affective disturbance. She was medicated with antipsychotic medications, but her condition continued to worsen into a classical catatonic presentation with serious medical complications confounded by what was thought to be possible neuroleptic malignant syndrome. However, after serum was shown to be “strongly positive” for anti-NMDA-receptor antibodies, a correct diagnosis of anti-NMDA-receptor encephalitis was made and appropriate treatment was initiated with intravenous methylprednisolone with a positive patient outcome [60].

The second patient, who presented because of urinary retention requiring catheterization attributed to anticholinergic effects of his medication, was a 42 year-old single male with moderate intellectual disability, autism and an “affective psychosis” that was in remission [60]. His medical presentation worsened with sepsis and decreased renal function. Behaviorally, the patient’s condition progressed to an acute delirious state. The confusing progression was eventually shown to be due to “positive anti-NMDA-receptor antibodies” and treatment was initiated for anti-NMDA-receptor encephalitis with two courses of methylprednisolone with gradual improvement over a period of a few months [60]. Although this is speculative, NMDA receptor hypofunction or disturbed function may confer a lower reserve of functional NMDA receptor mediated neurotransmission capability and, thus, greater vulnerability to deleterious consequences of NMDA receptor autoantibodies. Importantly, elevated levels of anti-NMDA-receptor antibodies are found in seemingly unaffected persons [61].

Of interest, type G immunoglobulin purified from a 53-year-old normally intelligent male with autism did not disrupt the translational movement of assembled NMDA receptors within the membranes of dissociated hippocampal neurons prepared from E18 Sprague Dawley rats [61]. The 53-year-old patient was shown to be seropositive for NMDA receptor autoantibodies using a cell-based assay that detected their binding to HEK293 cells transfected with the GluN1 and GluN2B-NMDA receptor subunits. However, normal translational movement of NMDA receptors within membranes of hippocampal neurons incubated with purified IgG from a seropositive patient with ASD, as measured quantitatively with synaptic mean square displacement and diffusion coefficient, does not reflect possible steric interference of the antibody with ligand binding and transduction of the glutamate signal. Importantly, for this study exploring the effect of anti-NMDA-receptor antibodies on the mobility of NMDA receptors within membranes of dissociated E18 rat hippocampal neurons, 24 patients with ASD and 18 healthy controls were screened for the presence of these autoantibodies; seropositivity was found in one of the 18 healthy controls free of any neurological or psychiatric disorder and only one of the 24 patients with ASD [61]. Thus, as noted above, the presence of anti-NMDA-receptor antibodies can occur without obvious pathogenic consequence. Similarly, the presence of anti-NMDA-receptor antibodies probably accounts for and/or contributes to a very small minority of cases presenting with ASD. However, in at least some patients, these autoantibodies serve as “experiments of nature” teaching us about acute consequences of targeting an extracellular epitope on the obligatory GluN1 subunit in “vulnerable” individuals. Perhaps, persons producing these autoantibodies with histories of ASD are at an increased risk for encephalitic consequences.

## 6. Medication Development of NMDA Receptor Modulators

**Figure 1:**
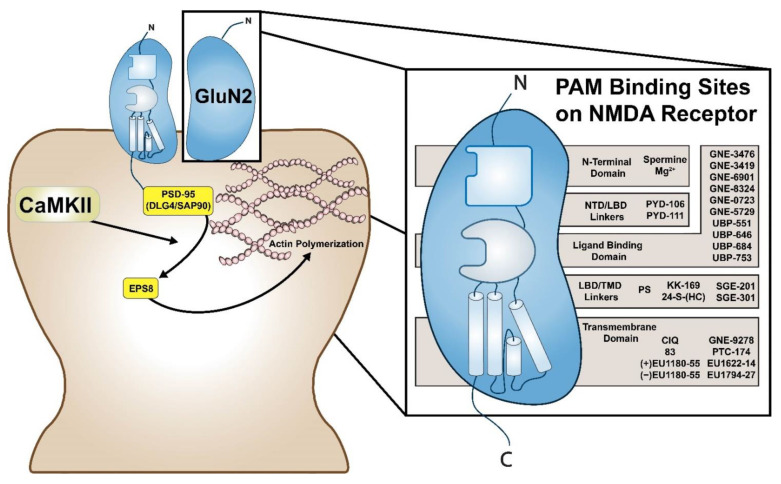
Intracellular signaling and allosteric modulatory binding sites on the NMDA receptor.

Possibilities for subtype-selective allosteric modulation of NMDA receptors result from the “tetrameric assembly and tight packing of NMDAR subunits” that “generate multiple protein/protein interfaces” [9] (Figure 1). The shared modular architecture of individual NMDA receptor subunits includes an extracellular region comprised of a clamshell-like amino terminal domain (NTD) and another clamshell-like ligand-binding domain (LBD); a transmembrane domain (TMD); and an intracellular C-terminal domain (CTD) [9]. The NTD is involved in receptor assembly and allosteric modulation, whereas the LBDs of GluN1 and GluN2 subunits are primarily involved in creation of binding sites for glycine/D-serine and glutamate, respectively. The ion channel pore of the NMDA receptor resides primarily within the TMDs of the four subunits and the CTD functions in receptor trafficking and intracellular signaling. The alignment of binding partners with the CTD in the complex architecture of the post-synaptic density of the excitatory synapse contributes to intracellular signaling and metabotropic functions of the NMDA receptor, many of which are not dependent on glutamate-gated cationic conductance [9,13]. As noted, the tight packing of the subunits lends itself to strong allosteric coupling of the receptor’s three regions: extracellular, TMD, and CTD [9]. For example, allosterically modulated conformational changes of the NTDs can affect channel activity. The shared motifs of the TMD of each individual subunit is comprised of three helical transmembrane segments (M1, M3, and M4) and the M2 re-entrant loop. The four M2 segments contribute to the channel’s ion selectivity and the four M3 segments contribute to the channel’s gating function. Allosteric modulation of the NTDs facilitate alternations between compact and relaxed conformations and, thereby, “stabilize high and low channel open probability states” [9].

The figure depicts the complex intracellular functioning of the NMDA receptor at the C-terminal domain leading to actin polymerization via EPS8 (left, see text for details). The right of the figure shows NMDA receptor binding sites of known GluN2 allosteric modulators; for comprehensive review see recent publication by Geoffrey and colleagues [9]. Figure was adapted from Burket et al., 2019; [13].

Naturally occurring polyamines (e.g., spermine and spermidine) and neurosteroids (e.g., pregnenolone sulfate (PS)) act as allosteric modulators of NMDA receptors. At micromolar concentrations, polyamines have selectivity for potentiating GluN2B-subtype-containing receptors, whose potentiation can be influenced by pH; at lower extracellular pH associated with ischemia and traumatic brain injury, potentiation by polyamines is enhanced [9]. Within a range of about 20–30 µM, PS has dominant PAM effects on both GluN2A- and GluN2B-subtype-containing NMDA receptors and at 5–10-fold higher micromolar concentrations a predominant NAM effect on GluN2C and GluN2D-containing NMDA receptors. Sensitivity to the pH dependence of PS-potentiation differs between GluN2A (i.e., stronger effect at lower pH), which is not observed with GluN2B-containing receptors [9].

### 6.1. Sodium Benzoate

Sodium benzoate, an inhibitor of D-amino acid oxidase (DAAO), has emerged as a useful tool in “proof of concept/principle” clinical trials designed to increase D-serine concentrations, the obligatory NMDA receptor co-agonist at many critical synaptically located NMDA receptors. Sodium benzoate enjoys a well-established safety profile and is used commercially as a food preservative. A twelve-week, open label pilot investigation explored effects of fixed daily doses of sodium benzoate adjusted for weight (<15 kg of body weight = 250 mg/day and ≥15 mg of body weight = 500 mg/day) combined with formal parent-administered “communication training” in six children with ASD (five boys, one girl; age range = 3 years and 7 months–9 years and 10 months) [62]. The children met DSM-5 diagnostic criteria for ASD and an additional inclusionary criterion of “severe communication problem”. Parents provided children with 40 min per day of formal communication training with the Chinese version of the “Core Vocabulary Communication System”, which is a picture vocabulary system requiring the subjects to either point to the picture corresponding to its spoken word or name the individual picture [62].

Developmental quotients were derived for each of the children from seven domains (i.e., gross motor, fine motor, comprehension, expressive language, situation-comprehension, personal-social, and self-help) and ranged from 24 to 56 at the beginning of the study, consistent with gross developmental delays [62]. The children were assessed every two weeks on a battery of outcome measures, some of which suggested treatment-related improvement (e.g., one child showed a 23-point improvement on his pre to post-test assessment of the developmental quotient, going from 56 to 79). Importantly, on a Clinical Global Impression of Improvement, an observer-rated 1–7 scale of global improvement, the communication skills of three subjects were rated as “much” improved [62]. There was also an impression that sodium benzoate had an “activating effect”, which may have been responsible for worsening the “hyperactivity” of two participants and adversely affecting the sleep of another. Clearly, it is difficult to resolve a therapeutic signal or attribute observed therapeutic signals to the medication in this pilot investigation for any individual subject; undoubtedly, the intense daily communication exercise between parent and child contributed to outcomes. Nonetheless, the authors argued that this pilot investigation should encourage future clinical trials. However, from a theoretical perspective, although increasing D-serine levels at the synapse does have greater selectivity for NMDA receptors than targeting the orthosteric glutamate binding site, intervening with GluN2A and GluN2B-subtype-selective PAMs would be predicted to better mimic physiological activation of these receptors while avoiding possible changes in receptor sensitivity and undesired off-target effects [13].

### 6.2. PAM Specific for GluN2A-Subtype-Containing Receptors

Genetech performed a high throughput screening of 1.4 million compounds and discovered GNE-3476, a “hit” that is a PAM specific for GluN2A-subtype-containing receptors [9]. GNE-3476 is a thiazolopryrimidinone that potentiates GluN2A-NMDA receptor responses with an EC_50_ of 10.3 µM. The discovery of the GNE-3476 hit compound spawned a medicinal chemistry effort to develop more potent and selective positive GluN2A allosteric modulators, such as GNE-3419 (EC_50_ = 2.03 µM); −6901 (EC_50_ = 0.33 µM); −8324 (EC_50_ = 2.43 µM); −0723 (EC_50_ = 0.021 µM) and −5729 (EC_50_ = 0.037 µM). These compounds act primarily at the LBD of the GluN2A-containing receptors and many enjoy about ≥100 fold greater positive allosteric modulatory selectivity on a micromolar basis for GluN2A- than GluN2B-containing NMDA receptors [9]. In any event, medication development efforts for NMDA receptor-subtype selective PAMs and NAMs have great promise for the treatment of ASD and other DDs [9,10,11].

#### 6.2.1. GNE-0723

The developmentally regulated switch to increased expression of GluN2A receptor subunits relative to expression of GluN2B subunits in pyramidal neurons contributes to efficient NMDA receptor-mediated induction of long-term potentiation [12]. Expression of the GluN2B receptor subunit is developmentally regulated, its synaptic expression predominates in the earliest stages of cortical development relative to GluN2A, and its expression enjoys anatomic selectivity. This developmentally regulated increased expression of GluN2A, which enjoys “faster kinetics” than GluN2B subunits, is also associated with a change in the distribution of GluN2 receptor subunits between synaptic and extrasynaptic sites (i.e., GluN2A predominates within synaptic sites, whereas GluN2B receptors predominate in extrasynaptic sites) [12,13,18]. GluN2D- and GluN2A-containing NMDA receptors on the surface of fast-spiking, parvalbumin-expressing (FSPV+) GABAergic inhibitory interneurons regulate their inhibitory input onto assemblies of pyramidal output neurons, which synchronizes the oscillatory outputs of these assemblies [12,33,63,64]. Interference with the rapid firing of these FSPV+ neurons (e.g., with uncompetitive inhibitors, such as ketamine and MK-801 (dizocilpine)) leads to disinhibition of the pyramidal cell assemblies and their increased cortical excitability [12,63,65,66]. Moreover, although pyramidal neurons themselves express GluN2A-containing NMDA receptors on their surface, the rapid firing properties of the FSPV+ interneurons and their greater basal activation that result in higher proportions of their NMDA receptors relieved of Mg^2+^ blockade are thought to make these inhibitory interneurons more susceptible and sensitive to both short- and long-term effects of uncompetitive open-channel NMDA receptor antagonists [12,63,65,66].

The consequences of decreased FSPV+ inhibitory input onto assemblies of pyramidal neurons include “network hypersynchrony” and increased seizure susceptibility, alterations of their oscillatory outputs, and increased activation of their extrasynaptic GluN2B-containing NMDA receptors, the latter subtype-selective NMDA receptors may mediate neurotoxicity in Alzheimer’s disease and other neuropsychiatric disorders [12]. In fact, memantine’s therapeutic mechanism of action may include blockade of aberrantly activated extrasynaptic GluN2B-containing NMDA receptors. Dravet syndrome (DS) is a severe unremitting epileptic disorder with onset in infancy due to heterozygous expression of missense mutations and microdeletions (i.e., copy number variants) of *SCN1A*, the gene coding the voltage-gated sodium ion channel Na_v_1.1 that is expressed in the axon initial segment (AIS) of FSPV+ and somatostatin-expressing GABAergic inhibitory interneurons; these inhibitory interneurons are embryologically derived from the medial ganglion eminence [12]. Na_v_1.1 in the AIS is necessary for generating action potentials and haploinsufficient expression in DS will lead to increased and dysregulated firing of assemblies of pyramidal neurons, “network hypersynchrony” and increased seizure susceptibility, and alterations of their oscillatory outputs. Interestingly, expression of Na_v_1.1 is reported to be decreased in several mouse models of Alzheimer’s disease [12]. The diminished inhibitory tone provided by FSPV+ GABAergic inhibitory interneurons onto assemblies of pyramidal output neurons in DS, Alzheimer’s disease and, possibly, at least some presentations of ASD, among other neuropsychiatric disorders stimulated interest in selective activation of GluN2A-containing NMDA receptors via GluN2A-subtype selective positive allosteric modulators (PAMs) [12,15,16,67]. A rationale for this pharmacotherapeutic strategy in ASD specifically was the subject of a recent review [13]. Data describing positive therapeutic effects of low-dose GNE-0723, a high-potency GluN2A-subtype selective PAM with favorable pharmacokinetic properties, in in vitro and animal models of DS and Alzheimer’s disease will be briefly reviewed [12]. These data encourage optimism and support similar explorations of GluN2A-subtype selective PAMs in animal models of ASD. A plausible in vivo strategy for correction of diminished central inhibitory tone, which contributes to the pathogenesis of ASD and other neuropsychiatric disorders, by anatomically selective activation of synaptic NMDA receptors on inhibitory interneurons using a high-potency GluN2A subtype-selective PAM (i.e., M-8324) was explored [68]. GluN2A PAMs lack significant intrinsic activity of their own and only act to potentiate NMDA receptor-mediated Ca^2+^ conductance of activated receptors. Inhibitory interneurons may be more sensitive to potentiation by GluN2A subtype-selective PAMs because of higher ambient synaptic glutamate concentrations in their non-stimulated state, compared to excitatory neurons [46,68]. Additionally, GluN2A subtype-selective PAMs may better restore physiological patterns of inhibitory transmission because PAMs act with temporal and spatial precision only where and when endogenous synaptic “activating” threshold concentrations of agonist (glutamate) and co-agonist (glycine or D-serine) are achieved [13,68].

With respect to pharmacokinetic properties, single oral doses of 1, 3 and 10 mg/kg of GNE-0723 showed dose-linear plasma concentrations with unbound Cmax concentrations of 5, 12, and 46 nM, respectively. Moreover, clearance of the PAM in brain and plasma was “slow” as unbound concentrations in brain and plasma remained stable from 0.5 to 24 h after a 3 mg/kg dose. Positive allosteric modulatory effects of GNE-0723 were shown with whole-cell patch-clamp recordings obtained from CHO cell lines expressing GluN2A-containing NMDA receptors [12]. In these studies, a 1 µM concentration GNE-0723 enhanced peak current and slowed current deactivation in response to glutamate. GNE-0723 (0.3 and 1.0 µM) increased peak current and decay time of electrically evoked NMDA receptor-mediated EPSCs in whole cell recordings from both layer 5 pyramidal neurons and PV+ interneurons in coronal cortical slices obtained from 2- to 3-month old adult mice (recordings were made at a holding potential of −70 mV and in the presence of reduced Mg^2+^ (0.5 mM)) [12]. As noted by the authors [12], these data suggest that effects of GNE-0723 on neural circuits and behavioral outputs reflect contributions of in vivo potentiation of GluN2A-containing NMDA receptors expressed on both excitatory and inhibitory cells.

Wild type mice treated with (+)-MK-801, the active enantiomer, showed dose-dependent increases in locomotor activity and rearings; GNE-0723 suppressed locomotor activity (10 mg/kg) and rearings (6 and 10 mg/kg) elicited by (+)-MK-801 in these wild type mice. However, although basal ambulatory locomotor activity of GluN2A knockout (GluN2A^KO^) mice was increased relative to wild type littermates, GNE-0723 had no effect on increased ambulatory locomotor activity and rearings in the GluN2A^KO^ mice [12]. These data confirm that the in vivo effects of GNE-0723 are mediated by GluN2A-containing NMDA receptors.

Intracranial EEG recordings from wild type mice were obtained to assess the effects of GNE-0723 and (+)-MK-801 on brain network oscillatory activity in behaving wild type mice [12]. At the low 3 mg/kg dose, GNE-0723 reduced the power of low-frequency oscillations within the 12–20 Hz band, but did not affect delta (0.5–6 Hz), theta (6–10 Hz) or gamma (30–80 Hz) bands during active awake and NREM sleep. At the higher 10 mg/kg dose, GNE-0723 reduced the power across all the frequency bands during active wake and NREM brain states. However, (+)-MK-801, the uncompetitive NMDA receptor antagonist, increased the power of low frequency 12–20 oscillations, which were suppressed by GNE-0723. Importantly, increased 12–20 Hz oscillatory “power” is associated with “network hypersynchrony” and risk for epileptic activity. Thus, because GNE-0723, a GluN2A PAM, reduced the power of low-frequency oscillations within the 12–20 Hz band, it was studied in mouse models of Dravet syndrome (i.e., the *Scn1a*-KI mouse that carries a heterozygous loss-of-function knock-in mutation causing Na_v_1.1 haploinsufficiency) and Alzheimer’s disease (the J20 mouse that overexpresses human amyloid precursor protein with the Swedish and Indiana familial AD mutations), whose characterization included aberrant low-frequency power, network hypersynchrony and epileptiform discharges [12].

In these two mouse models (the *Scn1a*-KI mouse and J20 mouse) with spontaneous epileptiform discharges occurring more frequently during periods of low locomotor activity in a novel environment, the epileptiform discharges were associated with increased 12 to 20 Hz power. Thirty minutes after administration of the 3 mg/kg dose of GNE-0723, the power of the 12 to 20 Hz oscillatory frequency was reduced in both mouse models (and wild type controls). Moreover, the 3 mg/kg dose of GNE-0723 reduced spontaneous epileptiform discharges in both mouse models as well. Finally, treatment every other day of both mouse models with the 3 mg/kg dose of GNE-0723 for five weeks improved the cognitive performance of the J20 mouse model of Alzheimer’s disease in the Morris water maze task and the *Scn1*-KI mouse model in a contextual fear conditioning task [12]. Overall, the data lend further support to testing of GluN2A PAMs in complementary behavioral models of ASD.

#### 6.2.2. M-8324

M-8324, a GluN2A subtype-selective PAM, increased spontaneous and sound-evoked spiking of inhibitory neurons and decreased spiking of excitatory neurons, while increasing the signal to noise ratio (SNR), in the primary auditory cortex of mice [68]. In vitro electrophysiological recordings in mouse coronal frontal slices showed that M-8324 enhanced NMDA receptor-mediated spontaneous spiking of GABAergic neurons recorded from layer 2/3 of mouse primary auditory cortex; there was no potentiation of excitatory neurons by M-8324 [68]. Similar findings were obtained when in vivo recordings were conducted in layer 2/3 of exposed right primary auditory cortex using multi-electrode arrays with infusions of M-8324 into the lateral ventricle of anesthetized mice. Spike waveforms were used to distinguish in vivo recordings obtained from regular spiking (RS) predominantly excitatory neurons and fast spiking (FS) predominantly parvalbumin-positive inhibitory neurons. As noted, the frequency of spontaneous and sound-evoked spiking was significantly increased by M-8324 in inhibitory neurons with significant concomitant reductions of spiking in excitatory neurons. M-8324 also caused significant improvement of the SNR for the excitatory neurons, as defined by their evoked spike frequency over spontaneous spike frequency, which was attributed to the enhanced inhibition reflected in a reduced E/I ratio. Delayed toxic effects of exposure to loud noise (8 kHz, 115 dB for 2 h) appearing within 10 days in mice and related to the pathogenic development of the clinical syndrome of tinnitus in humans was prevented by infusion of M-8324 prior to noise exposure [68]. Toxic deleterious effects of loud noise exposure, which were not associated with hearing deficits, were attenuated by prior infusion of M-8324 and included changes in behavior (loud noise-induced change in the startle response ratio), histopathology (loud noise-induced significant reduction in PV-expressing neurons in layer 2/3 of primary auditory cortex) and electrophysiological recordings (loud noise-induced reductions in the decay time of spontaneous inhibitory post synaptic currents (sIPSC) recorded in excitatory neurons of primary auditory cortex) [68]. The latter data suggested that M-8324, a high-potency, highly selective GluN2A PAM that potentiates NMDA receptor-activated GABAergic transmission, may have protective effects on specific parvalbumin-expressing inhibitory interneurons. In addition to immediate therapeutic effects of GluN2A PAMs associated with their facilitating inhibitory input onto assemblies of cortical pyramidal output neurons, which, thereby, may improve synchronous oscillatory rhythms critical for higher executive and complex socio-cognitive functions, neuroprotective effects may also be conveyed by GluN2A PAMs [13,68]. In any event, a growing body of literature supports continued development and empirical clinical trials of selective GluN2A PAMs for the treatment of neurodevelopmental disorders, including ASD.

## 7. Conclusions

The high heritability of ASD encourages genotyping of affected persons and their first-degree relatives in order to identify candidate loci that may be associated with risk for, and/or contribute to, the pathogenesis of this complex illness phenotype. As shown in this selective Review, a shared pathophysiological consequence of absent or haploinsufficient expression of several seemingly unrelated risk alleles associated with ASD in relevant mouse models is disruption of NMDA receptor-mediated neurotransmission. The nature of the disruptions extend from NMDA receptor hypofunction (NRH) to pathogenic imbalance between activation mediated selectively between GluN2B- and GluN2A-subtype-containing NMDA receptors. However, pharmacotherapeutic correction of this disrupted balance is confounded due to differences in developmental, cell-type and anatomic regional expression of the four GluN2 genes, as well as the eight GluN1 alternatively spliced isoforms. Ultimately, gating properties of individual NMDA receptors expressed on the surface of specific cell types within specialized neural circuits that are provoked by socio-cognitive challenges influence behavioral outputs which, in the case of patients with ASD, may not be consistent with neurotypical subjects and deemed psychopathological. Because critical windows exist during which targeted therapeutic interventions may be best able to facilitate compensatory mechanisms to mitigate deleterious consequences of increased genetic risk, there is heightened interest in the earliest possible diagnosis of ASD. Ideally, genotyping would contribute to earlier diagnosis and, thereby, facilitate earlier initiation of effective disease-modifying interventions, resulting in improved social, educational and vocational trajectories of many affected individuals. In the case of genetic lesions with regionally, developmentally and cell-type-selective deleterious effects on NMDA receptor-mediated activation, especially disruptions that create imbalance between GluN2B and GluN2A-subtype-containing NMDA receptor-mediated neurotransmission, translationally developed therapeutic allosteric modulatory medications have great theoretical appeal.

Ideally, PAMs and NAMs developed as medications would lack intrinsic efficacy of their own and act with spatial and temporal selectivity to fine tune disrupted balance between glutamate-gated opening of and cationic conductance by GluN2B and GluN2A-NMDA receptors. Thus, therapeutic allosteric modulatory effects would act only where and when endogenous glutamate and its obligatory co-agonists are released in response to higher socio-cognitive challenges. PAMs and NAMs would also have lesser liability for causing off-target adverse effects and changing the sensitivity of the receptors’ orthosteric binding sites, which are liabilities associated with administering medications that act as competitive agonists and antagonists at glutamate agonist binding sites. Salient aspects of the electronic configurations of orthosteric sites that bind glutamate are shared diffusely across the brain by many classes and subtypes of ionotropic and metabotropic glutamate receptors, which is why they may not afford the target selectivity to address deficits in higher socio-cognitive functions and are more likely to cause off-target side effects and receptor desensitization upon chronic administration. In any event, it is hoped that NMDA receptor-subtype-selective PAMs and NAMs will be developed as medications that can be studied in predictive mouse models of ASD and safely brought to the clinic, to promote better social and vocational outcomes when administered in the context of interdisciplinary, multimodal individualized treatment plans.

## Data Availability

Not applicable.
